# Supplementary material for: Tumor phylogeography reveals block-shaped spatial heterogeneity and the mode of evolution in Hepatocellular Carcinoma
Source: Nat Commun. 2024 Apr 12;15:3169. doi: 10.1038/s41467-024-47541-9 (PMC11015015; doi:10.1038/s41467-024-47541-9)
Supplement: Supplementary file 1 — Supplementary Information [file 41467_2024_47541_MOESM1_ESM.pdf]

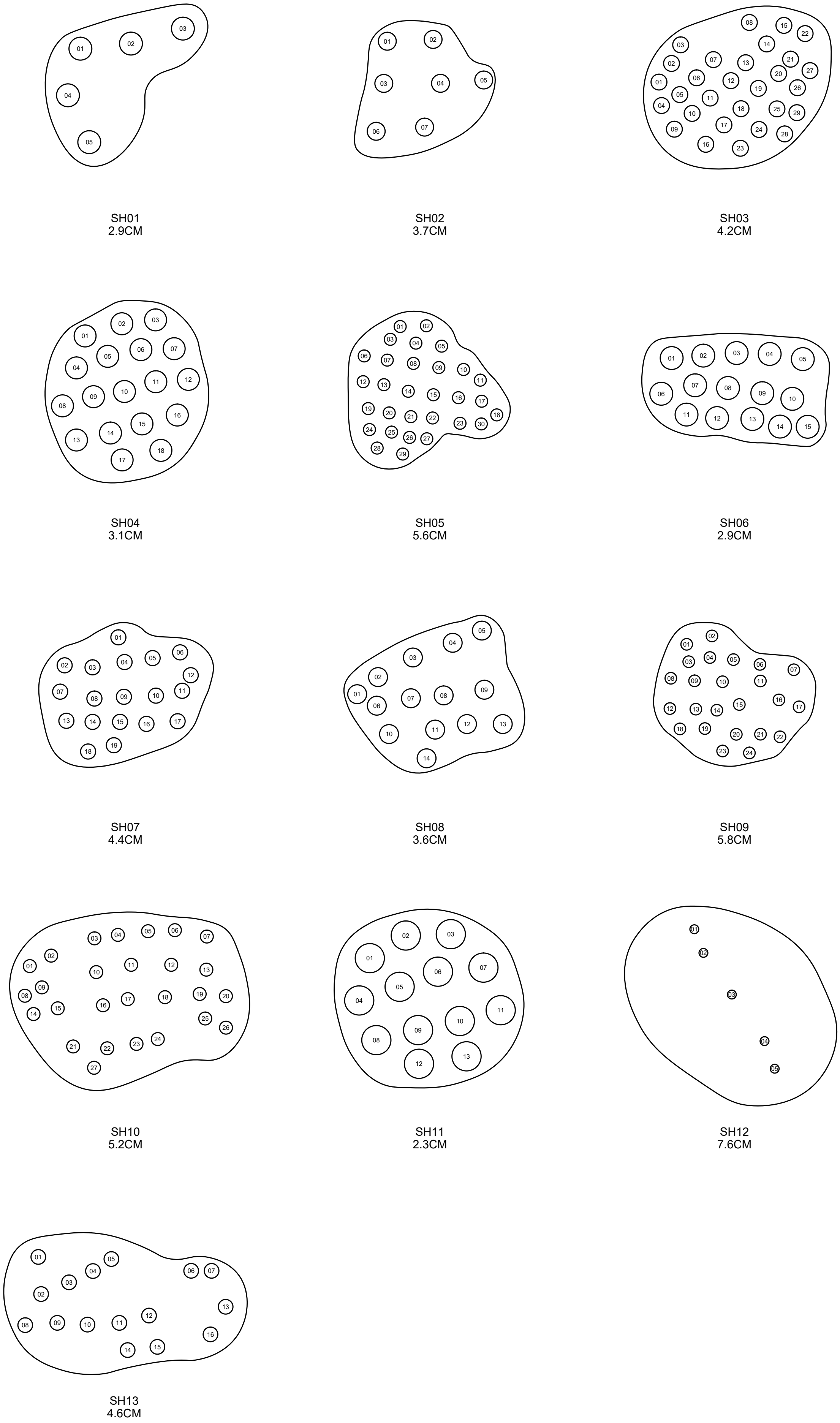

Supplementary Figure 1: The spatial sampling of tumor sectors. The physical locations and shapes of the tumors were recorded after taking multiple sectors from the tumor.

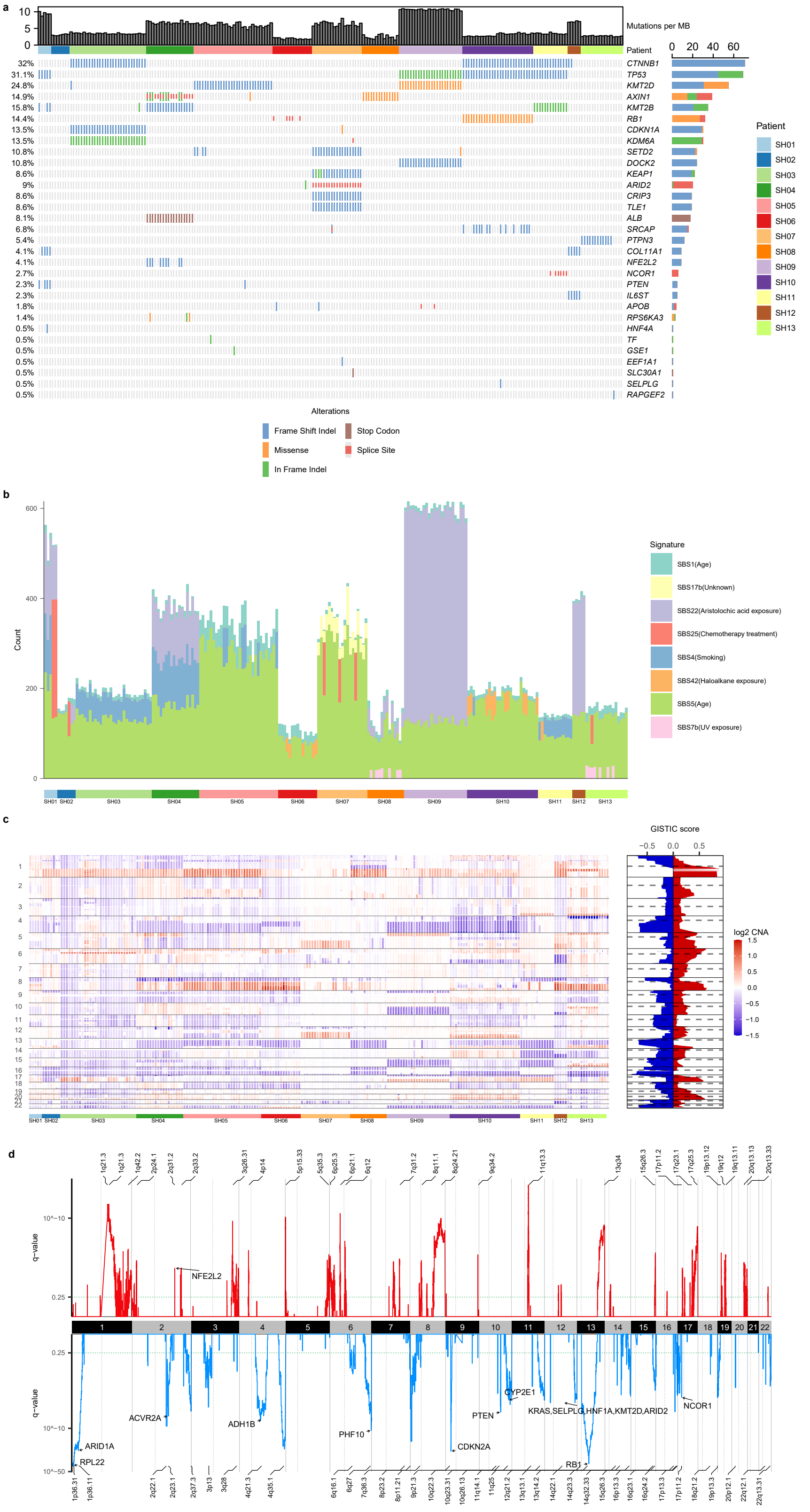

Supplementary Figure 2: The genomic landscape of the patient cohort. a) The landscape of driver genes across all the samples, the top bar on the oncoprint plot indicates the TMB of the samples. The list of driver genes was taken from a recent integrative analysis of HCC genomes (https://www.thno.org/v12p4703). b) The mutational signatures of the samples. c) The copy number landscape of the tumor samples. The GISTIC output of the current cohort was plotted to the right-handside of the plot. d) the GISTIC output of the TCGA LIHC cohort were plotted (see Methods). Source data are provided as a Source Data file.

Supplementary Figure 3: The genomic characterization of the patient cohort (page 3-15 , see figure legend on page 15).

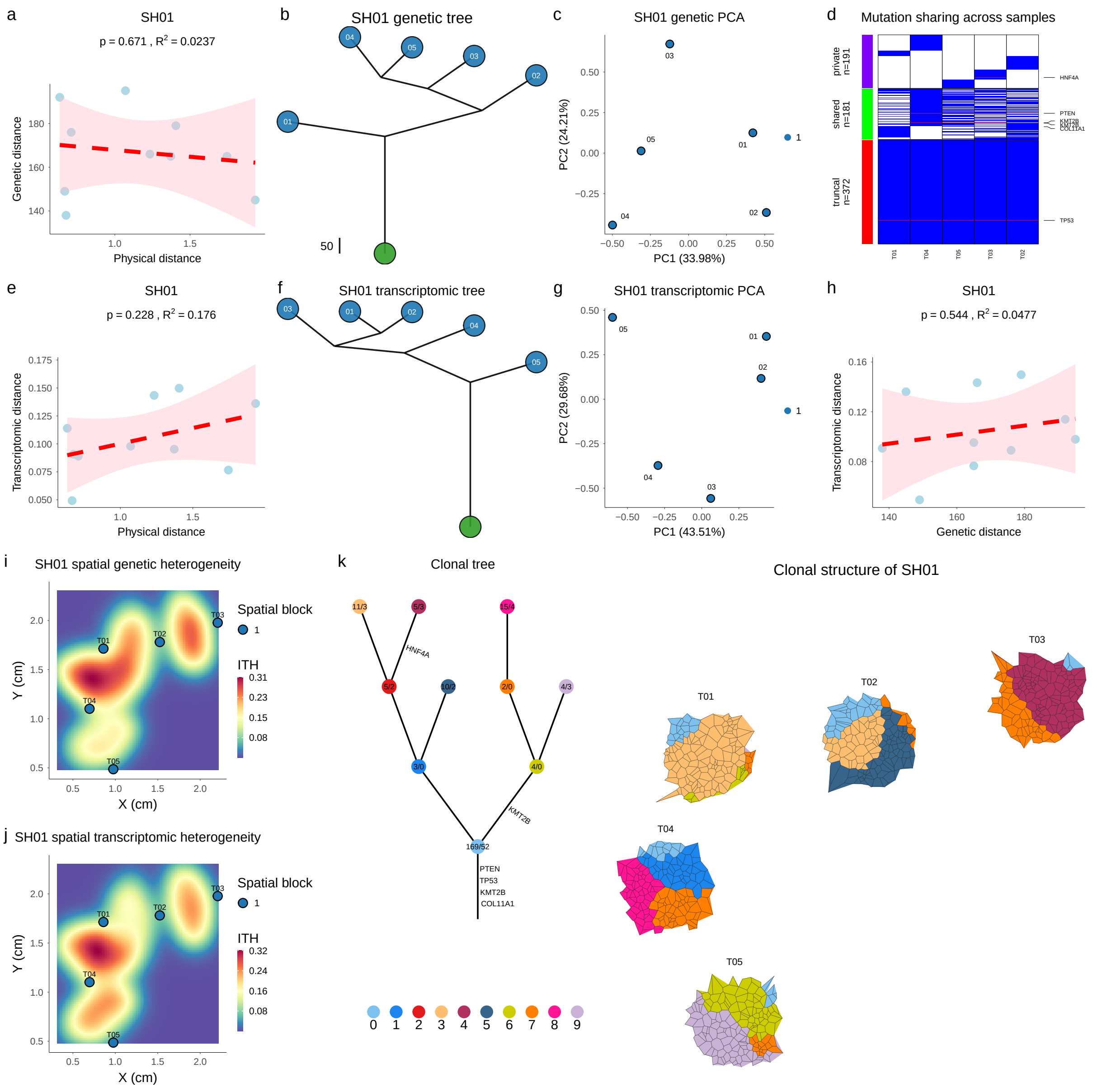

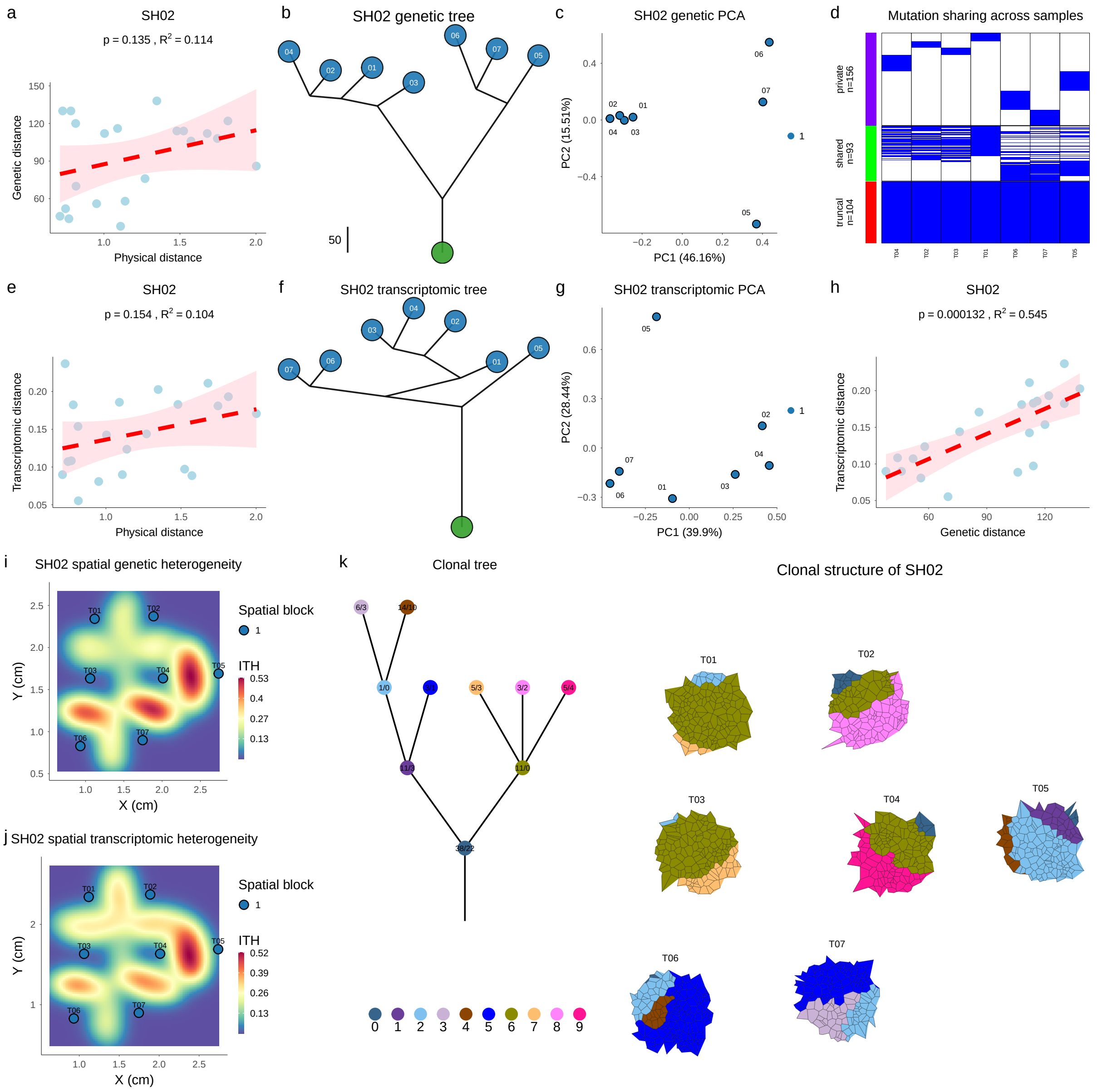

a
SH02
p = 0.135 , R2 = 0.114
Genetic distance
Physical distance
b
SH02 genetic tree
c
SH02 genetic PCA
PC1 (46.16%)
PC2 (15.51%)
d
Mutation sharing across samples
private n=156
shared n=93
truncal n=104
e
SH02
p = 0.154 , R2 = 0.104
Transcriptomic distance
Physical distance
f
SH02 transcriptomic tree
g
SH02 transcriptomic PCA
PC1 (39.9%)
PC2 (28.44%)
h
SH02
p = 0.000132 , R2 = 0.545
Transcriptomic distance
Genetic distance
i
SH02 spatial genetic heterogeneity
Spatial block
ITH
X (cm)
Y (cm)
j
SH02 spatial transcriptomic heterogeneity
Spatial block
ITH
X (cm)
Y (cm)
k
Clonal tree
Clonal structure of SH02

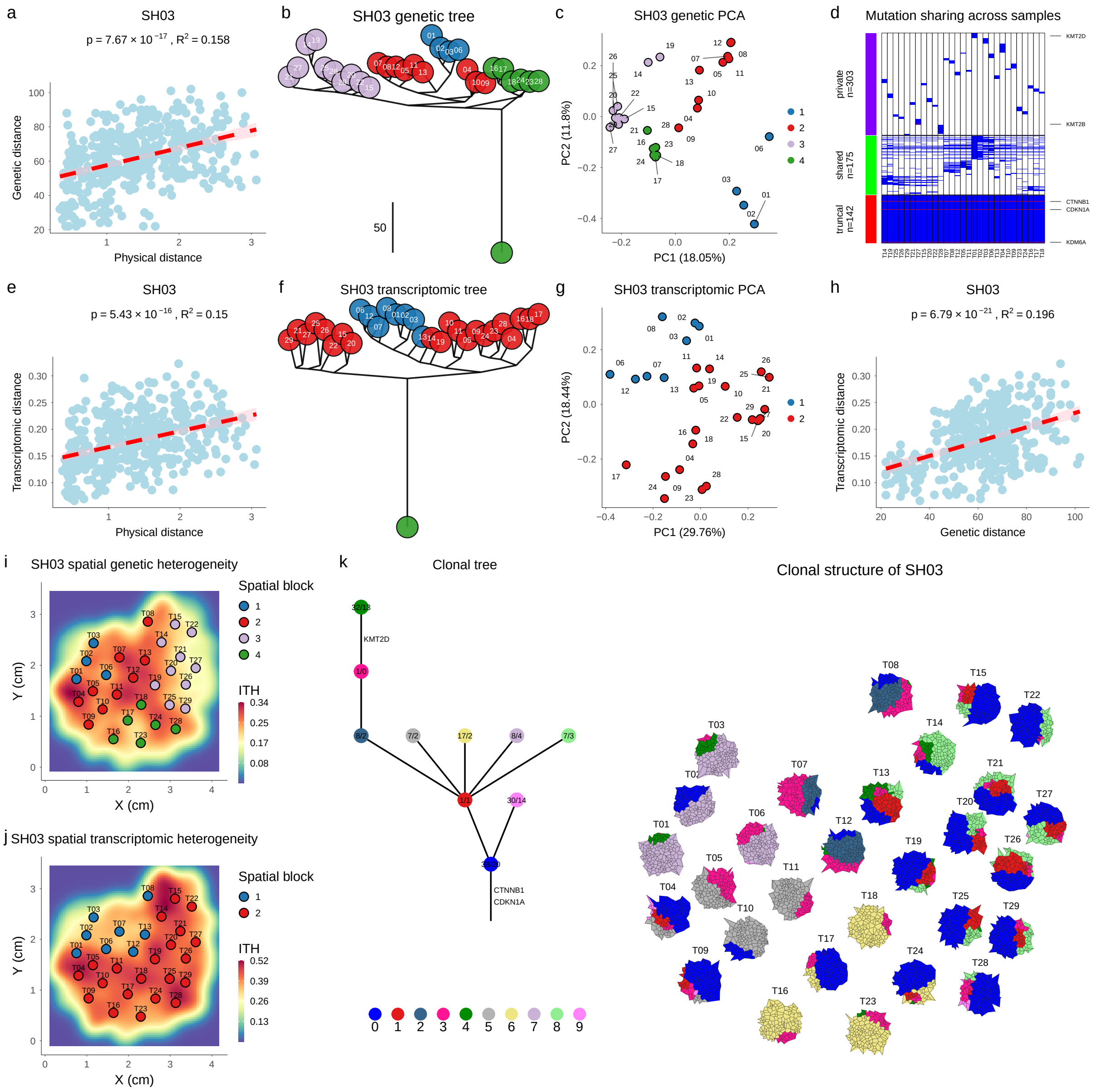
SH03
SH03 genetic tree
SH03 genetic PCA
Mutation sharing across samples
SH03 transcriptomic tree
SH03 transcriptomic PCA
SH03 spatial genetic heterogeneity
SH03 spatial transcriptomic heterogeneity
Clonal tree
Clonal structure of SH03
Physical distance
Genetic distance
Transcriptomic distance
Spatial block
ITH
KMT2D
CTNNB1
CDKN1A
KDM6A
KMT2B

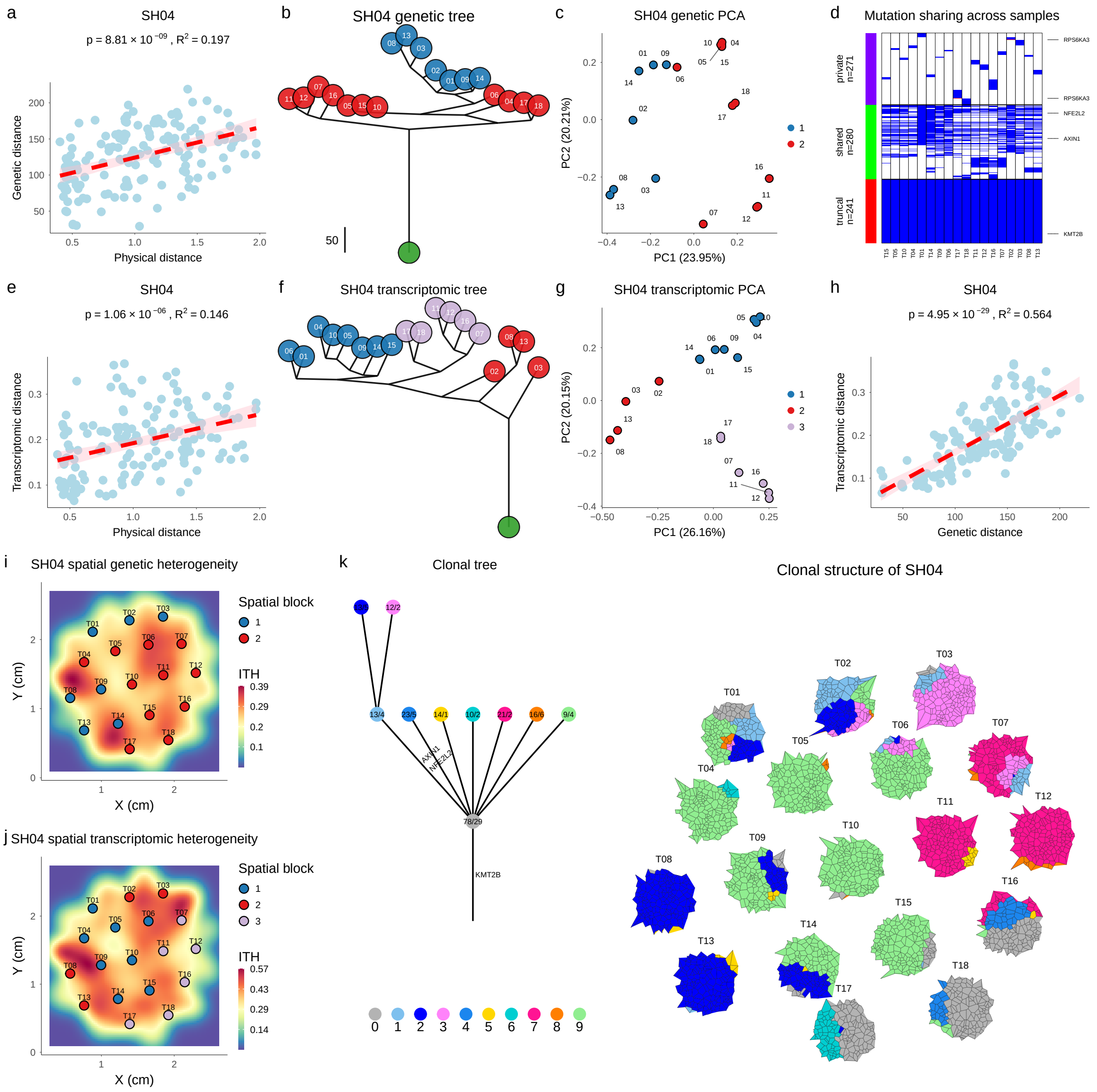
a
SH04
p = 8.81 × 10^−09, R² = 0.197
Genetic distance
Physical distance
b
SH04 genetic tree
c
SH04 genetic PCA
PC2 (20.21%)
PC1 (23.95%)
d
Mutation sharing across samples
private n=271
shared n=280
truncal n=241
RPS6KA3
NFE2L2
AXIN1
KMT2B
e
SH04
p = 1.06 × 10^−06, R² = 0.146
Transcriptomic distance
Physical distance
f
SH04 transcriptomic tree
g
SH04 transcriptomic PCA
PC2 (20.15%)
PC1 (26.16%)
h
SH04
p = 4.95 × 10^−29, R² = 0.564
Transcriptomic distance
Genetic distance
i
SH04 spatial genetic heterogeneity
Spatial block
ITH
Y (cm)
X (cm)
j
SH04 spatial transcriptomic heterogeneity
Spatial block
ITH
Y (cm)
X (cm)
k
Clonal tree
AXIN1
NFE2L2
KMT2B
Clonal structure of SH04

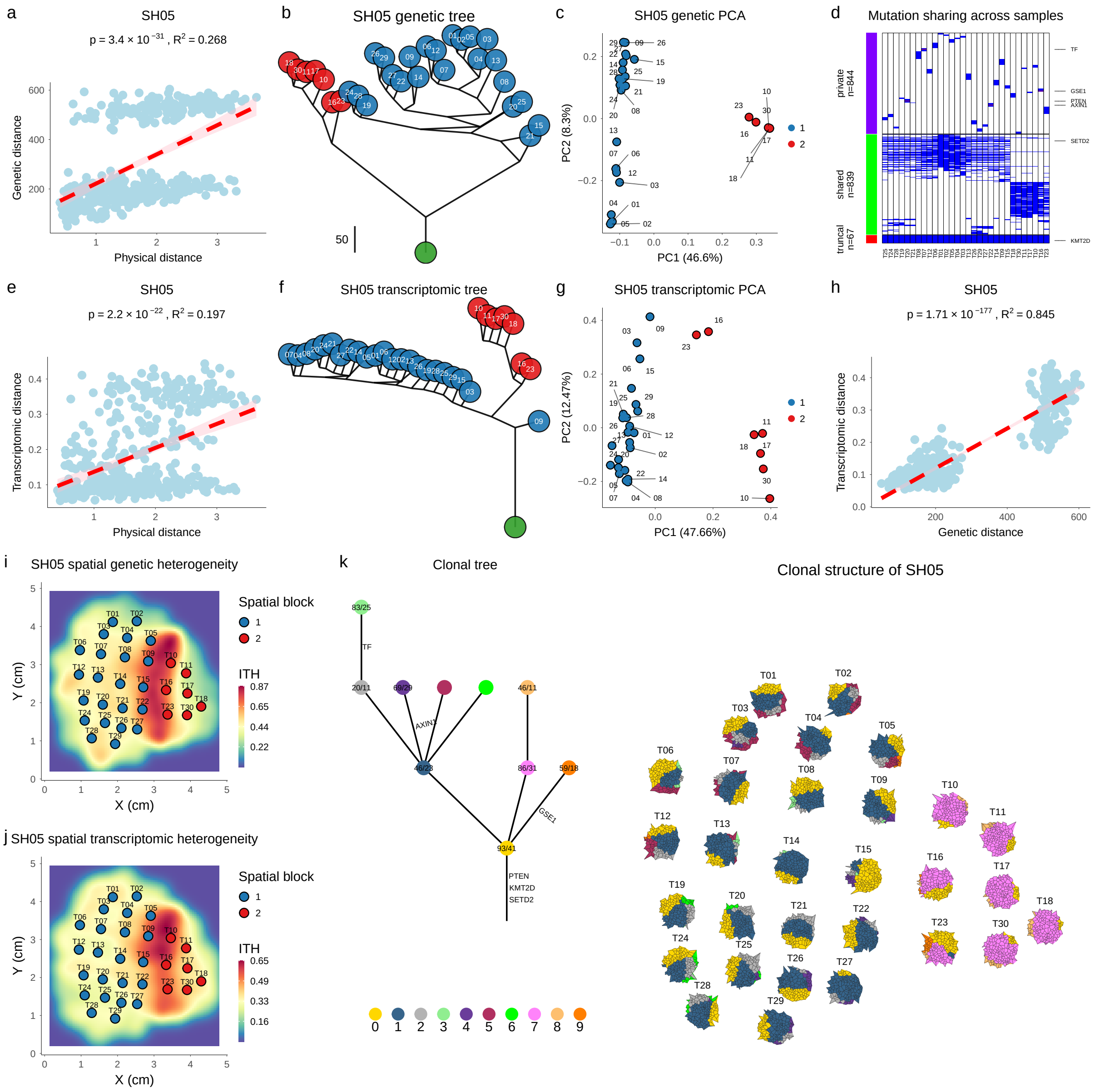
a
SH05
p = 3.4 × 10^−31, R^2 = 0.268
Genetic distance
Physical distance
b
SH05 genetic tree
50
c
SH05 genetic PCA
PC1 (46.6%)
PC2 (8.3%)
d
Mutation sharing across samples
private n=844
shared n=839
truncal n=67
TF
GSE1
PTEN
AXIN1
SETD2
KMT2D
e
SH05
p = 2.2 × 10^−22, R^2 = 0.197
Transcriptomic distance
Physical distance
f
SH05 transcriptomic tree
g
SH05 transcriptomic PCA
PC1 (47.66%)
PC2 (12.47%)
h
SH05
p = 1.71 × 10^−177, R^2 = 0.845
Transcriptomic distance
Genetic distance
i
SH05 spatial genetic heterogeneity
Spatial block
ITH
X (cm)
Y (cm)
j
SH05 spatial transcriptomic heterogeneity
Spatial block
ITH
X (cm)
Y (cm)
k
Clonal tree
TF
AXIN1
GSE1
PTEN
KMT2D
SETD2
Clonal structure of SH05

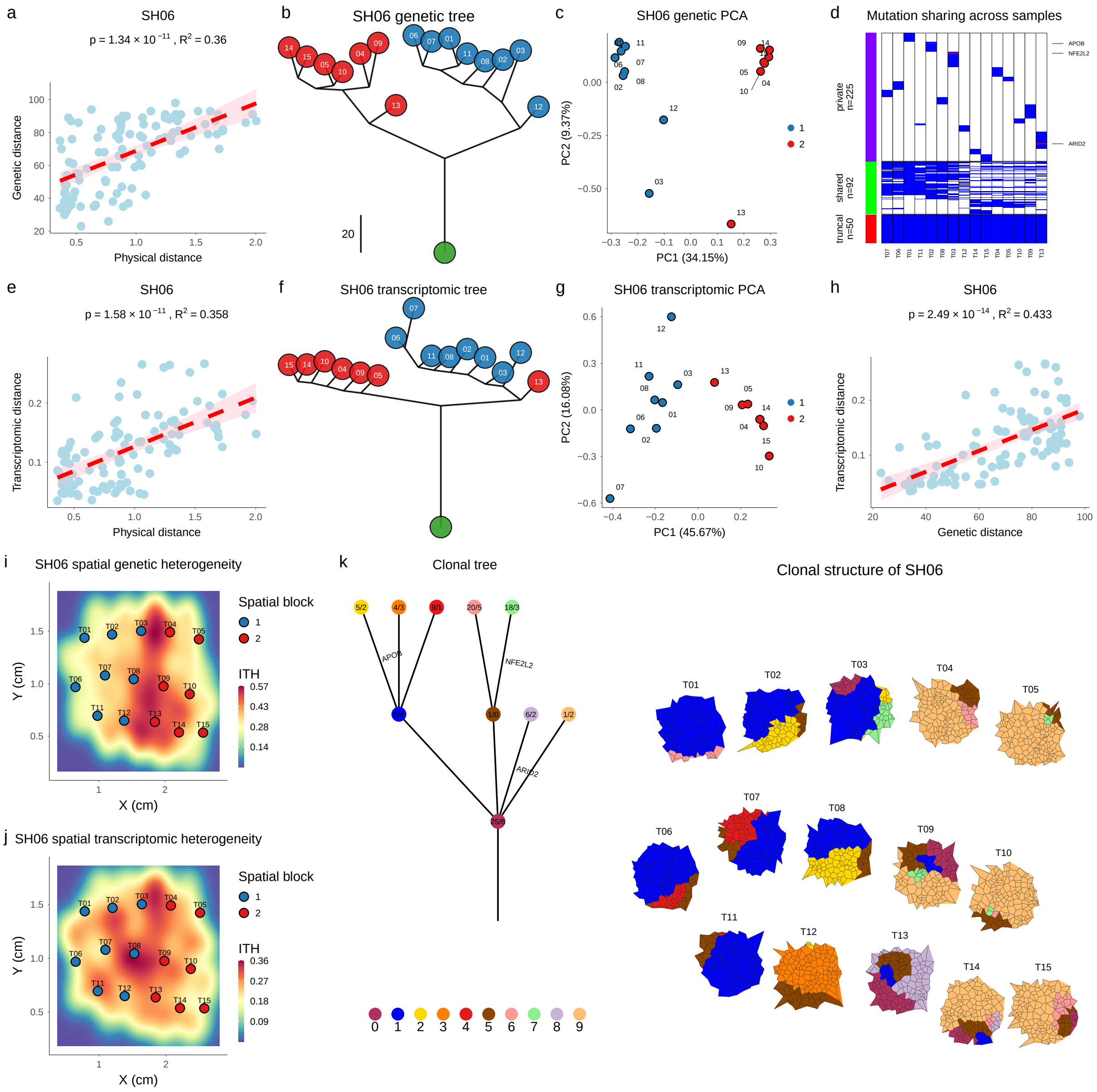

a
SH06
p = 1.34 × 10^−11, R² = 0.36
Genetic distance
Physical distance
b
SH06 genetic tree
c
SH06 genetic PCA
PC1 (34.15%)
PC2 (9.37%)
d
Mutation sharing across samples
private n=225
shared n=92
truncal n=50
APOB
NFE2L2
ARID2
e
SH06
p = 1.58 × 10^−11, R² = 0.358
Transcriptomic distance
Physical distance
f
SH06 transcriptomic tree
g
SH06 transcriptomic PCA
PC1 (45.67%)
PC2 (16.08%)
h
SH06
p = 2.49 × 10^−14, R² = 0.433
Transcriptomic distance
Genetic distance
i
SH06 spatial genetic heterogeneity
Spatial block
ITH
Y (cm)
X (cm)
j
SH06 spatial transcriptomic heterogeneity
Spatial block
ITH
Y (cm)
X (cm)
k
Clonal tree
APOB
NFE2L2
ARID2
Clonal structure of SH06

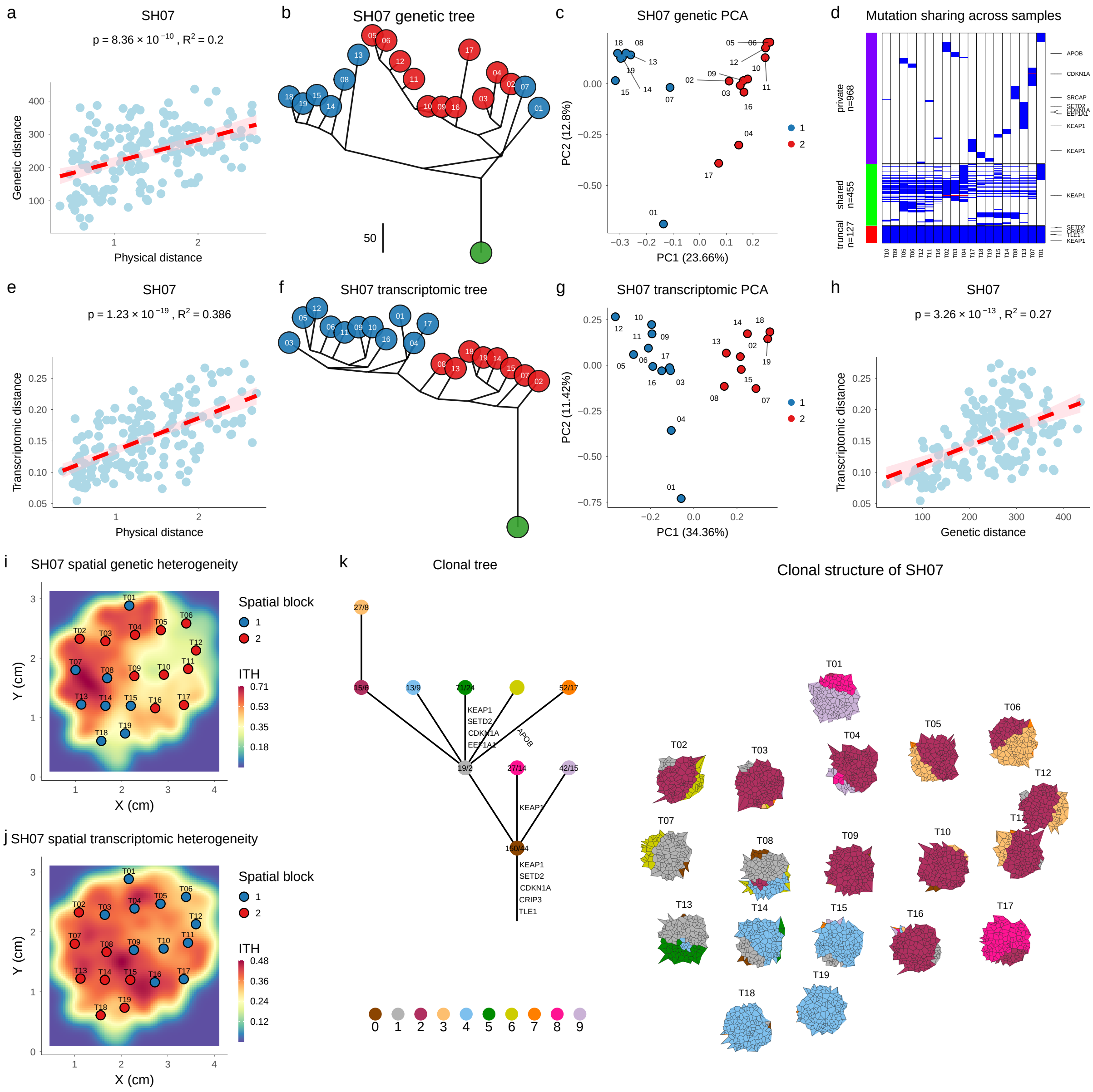

a
SH07
p = 8.36 × 10^−10 , R^2 = 0.2
Genetic distance
Physical distance
b
SH07 genetic tree
50
c
SH07 genetic PCA
PC2 (12.8%)
PC1 (23.66%)
d
Mutation sharing across samples
private n=968
shared n=455
truncal n=127
APOB
CDKN1A
SRCAP
SETD2
KEAP1
e
SH07
p = 1.23 × 10^−19 , R^2 = 0.386
Transcriptomic distance
Physical distance
f
SH07 transcriptomic tree
g
SH07 transcriptomic PCA
PC2 (11.42%)
PC1 (34.36%)
h
SH07
p = 3.26 × 10^−13 , R^2 = 0.27
Transcriptomic distance
Genetic distance
i
SH07 spatial genetic heterogeneity
Spatial block
ITH
Y (cm)
X (cm)
j
SH07 spatial transcriptomic heterogeneity
Spatial block
ITH
Y (cm)
X (cm)
k
Clonal tree
KEAP1
SETD2
CDKN1A
EEF1A1
APOB
KEAP1
KEAP1
SETD2
CDKN1A
CRIP3
TLE1
Clonal structure of SH07

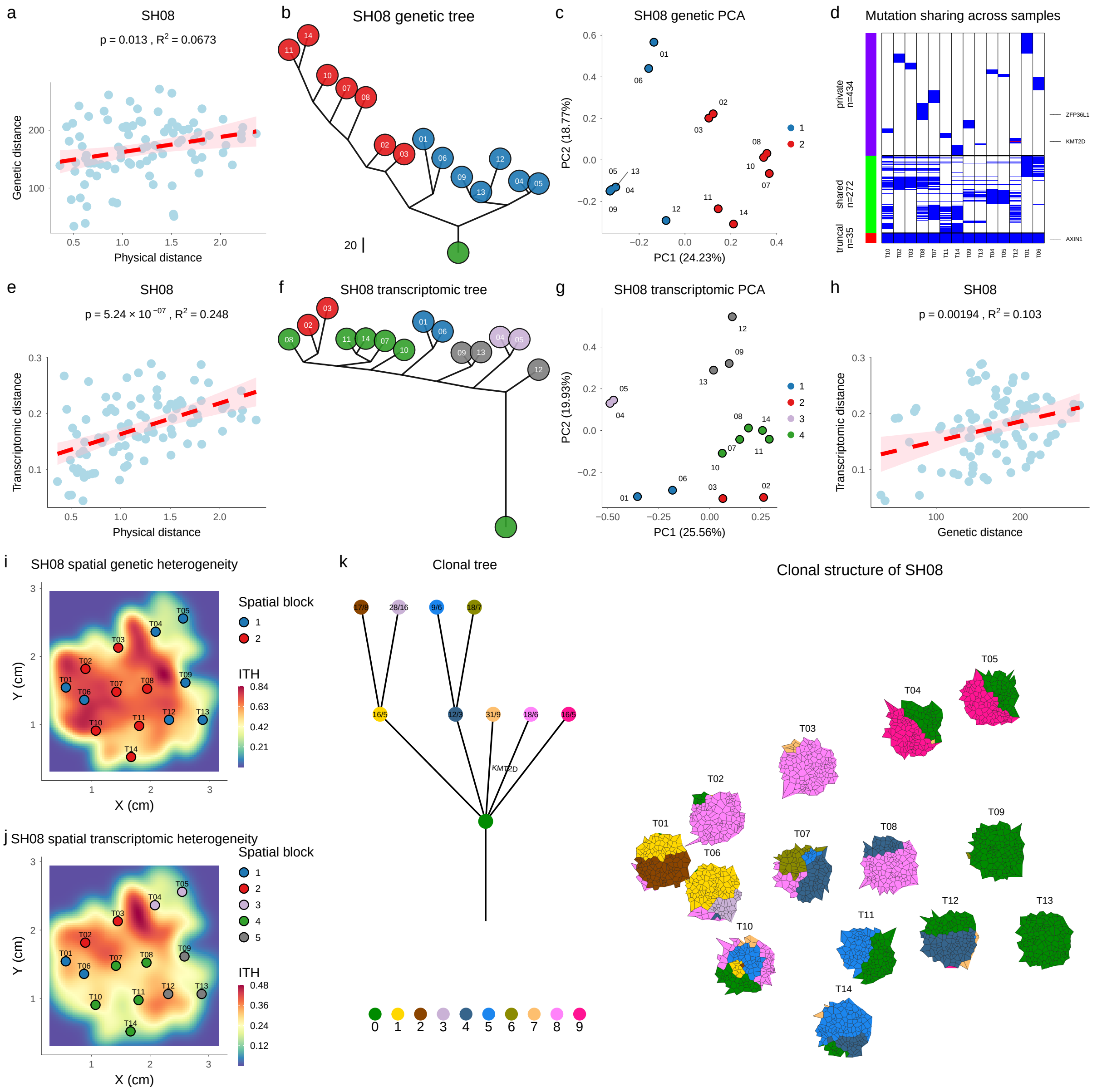

a
SH08
p = 0.013 , R2 = 0.0673
Genetic distance
Physical distance
b
SH08 genetic tree
c
SH08 genetic PCA
PC1 (24.23%)
PC2 (18.77%)
d
Mutation sharing across samples
private n=434
shared n=272
truncal n=35
ZFP36L1
KMT2D
AXIN1
e
SH08
p = 5.24 × 10−07 , R2 = 0.248
Transcriptomic distance
Physical distance
f
SH08 transcriptomic tree
g
SH08 transcriptomic PCA
PC1 (25.56%)
PC2 (19.93%)
h
SH08
p = 0.00194 , R2 = 0.103
Transcriptomic distance
Genetic distance
i
SH08 spatial genetic heterogeneity
Spatial block
ITH
X (cm)
Y (cm)
j
SH08 spatial transcriptomic heterogeneity
Spatial block
ITH
X (cm)
Y (cm)
k
Clonal tree
KMT2D
Clonal structure of SH08

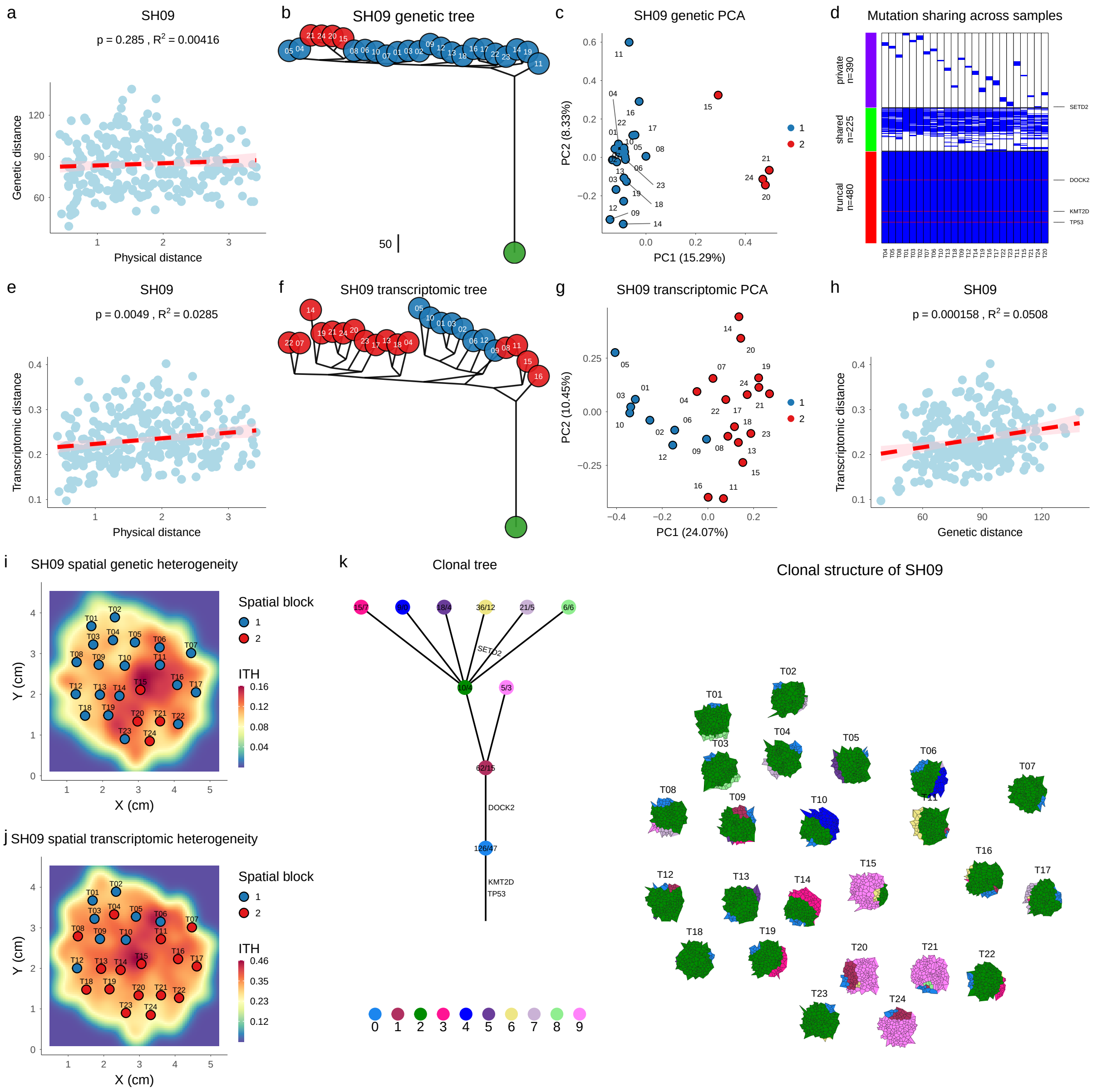
a
SH09
p = 0.285 , R2 = 0.00416
Genetic distance
Physical distance
b
SH09 genetic tree
c
SH09 genetic PCA
PC1 (15.29%)
PC2 (8.33%)
d
Mutation sharing across samples
private n=390
shared n=225
truncal n=480
SETD2
DOCK2
KMT2D
TP53
e
SH09
p = 0.0049 , R2 = 0.0285
Transcriptomic distance
Physical distance
f
SH09 transcriptomic tree
g
SH09 transcriptomic PCA
PC1 (24.07%)
PC2 (10.45%)
h
SH09
p = 0.000158 , R2 = 0.0508
Transcriptomic distance
Genetic distance
i
SH09 spatial genetic heterogeneity
Spatial block
ITH
X (cm)
Y (cm)
j
SH09 spatial transcriptomic heterogeneity
Spatial block
ITH
X (cm)
Y (cm)
k
Clonal tree
SETD2
DOCK2
KMT2D
TP53
Clonal structure of SH09

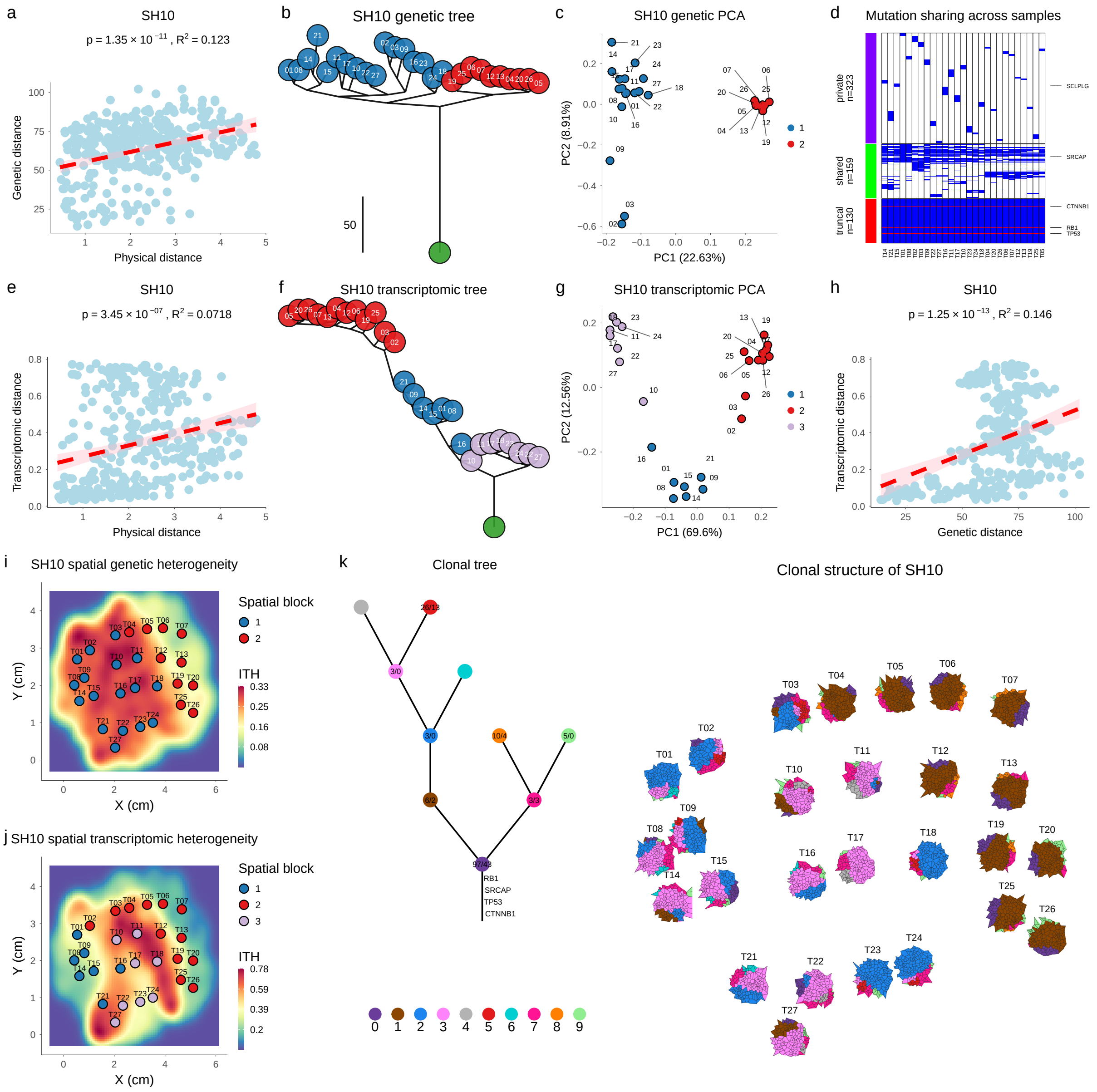
a
SH10
p = 1.35 × 10^−11 , R² = 0.123
Genetic distance
Physical distance
b
SH10 genetic tree
50
c
SH10 genetic PCA
PC2 (8.91%)
PC1 (22.63%)
d
Mutation sharing across samples
private n=323
shared n=159
truncal n=130
SELPLG
SRCAP
CTNNB1
RB1
TP53
e
SH10
p = 3.45 × 10^−07 , R² = 0.0718
Transcriptomic distance
Physical distance
f
SH10 transcriptomic tree
g
SH10 transcriptomic PCA
PC2 (12.56%)
PC1 (69.6%)
h
SH10
p = 1.25 × 10^−13 , R² = 0.146
Transcriptomic distance
Genetic distance
i
SH10 spatial genetic heterogeneity
Spatial block
ITH
Y (cm)
X (cm)
j
SH10 spatial transcriptomic heterogeneity
Spatial block
ITH
Y (cm)
X (cm)
k
Clonal tree
RB1
SRCAP
TP53
CTNNB1
Clonal structure of SH10

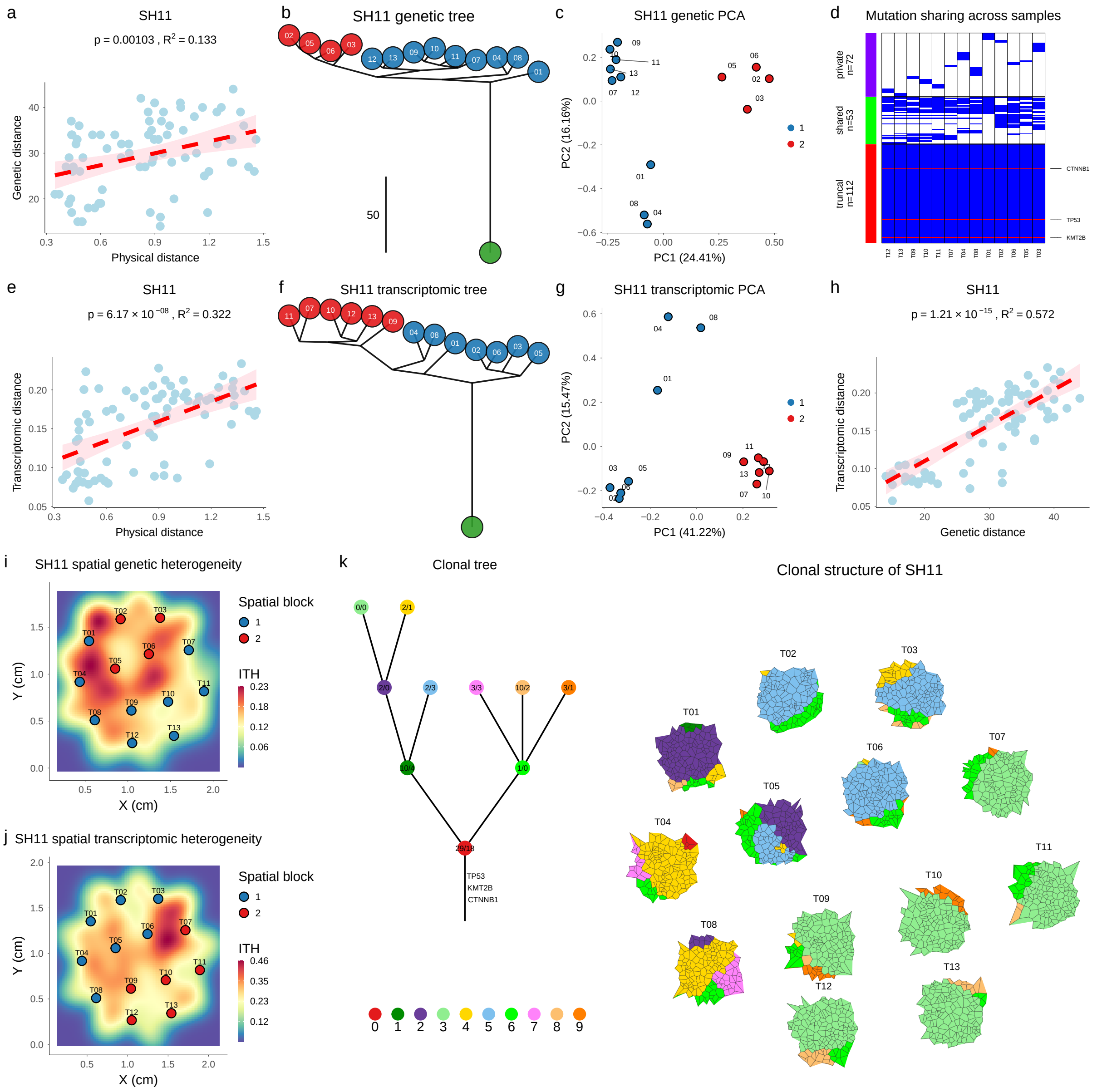
a
SH11
p = 0.00103 , R2 = 0.133
Genetic distance
Physical distance
b
SH11 genetic tree
50
c
SH11 genetic PCA
PC2 (16.16%)
PC1 (24.41%)
d
Mutation sharing across samples
private n=72
shared n=53
truncal n=112
CTNNB1
TP53
KMT2B
e
SH11
Transcriptomic distance
Physical distance
f
SH11 transcriptomic tree
g
SH11 transcriptomic PCA
PC2 (15.47%)
PC1 (41.22%)
h
SH11
p = 1.21 × 10−15 , R2 = 0.572
Transcriptomic distance
Genetic distance
i
SH11 spatial genetic heterogeneity
Spatial block
ITH
Y (cm)
X (cm)
j
SH11 spatial transcriptomic heterogeneity
Spatial block
ITH
Y (cm)
X (cm)
k
Clonal tree
TP53
KMT2B
CTNNB1
Clonal structure of SH11

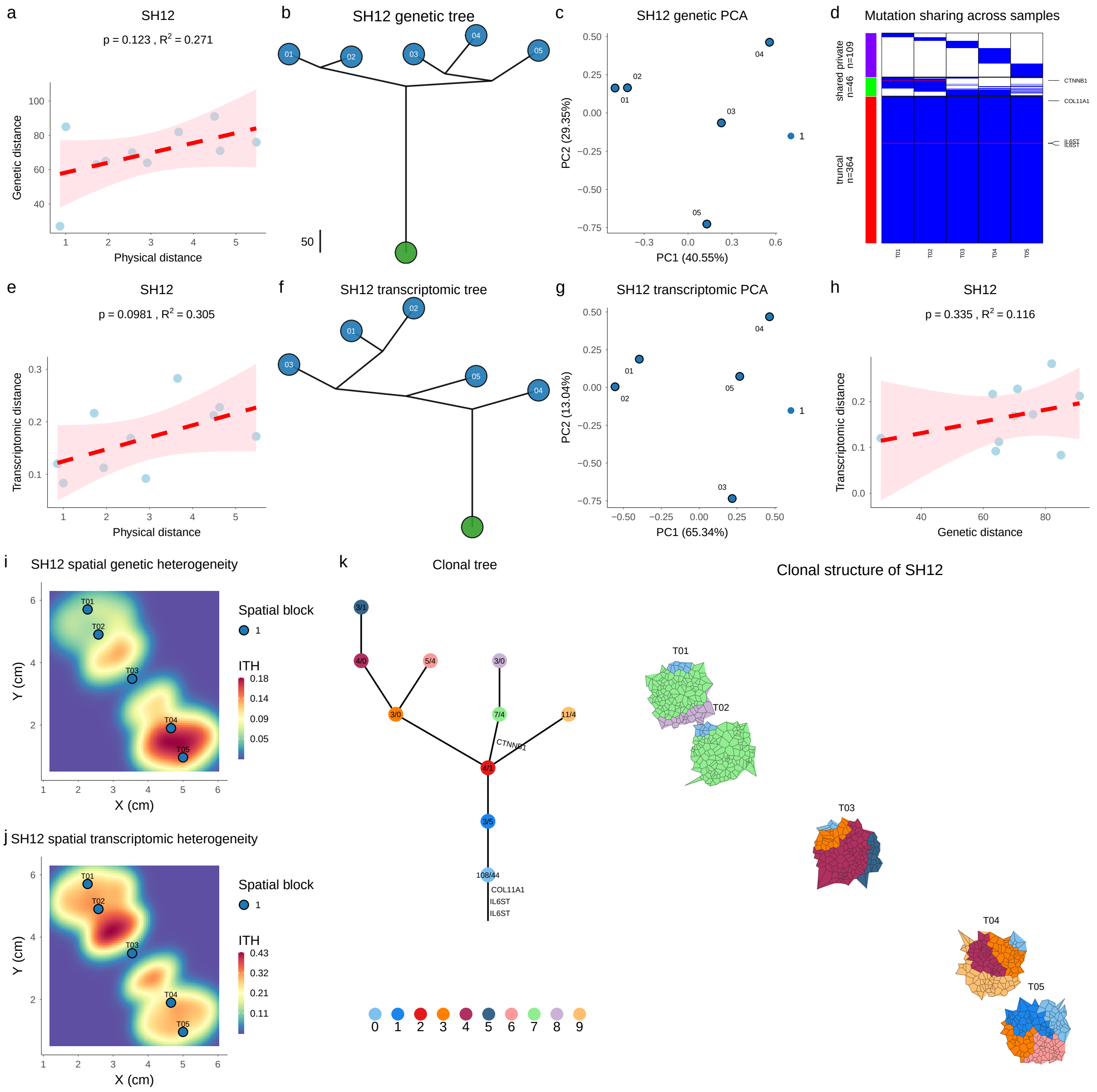

a
SH12
p = 0.123 , R2 = 0.271
Genetic distance
Physical distance
b
SH12 genetic tree
c
SH12 genetic PCA
PC2 (29.35%)
PC1 (40.55%)
d
Mutation sharing across samples
shared private n=109
n=46
truncal n=364
CTNNB1
COL11A1
e
SH12
p = 0.0981 , R2 = 0.305
Transcriptomic distance
Physical distance
f
SH12 transcriptomic tree
g
SH12 transcriptomic PCA
PC2 (13.04%)
PC1 (65.34%)
h
SH12
p = 0.335 , R2 = 0.116
Transcriptomic distance
Genetic distance
i
SH12 spatial genetic heterogeneity
Spatial block
ITH
Y (cm)
X (cm)
j
SH12 spatial transcriptomic heterogeneity
Spatial block
ITH
Y (cm)
X (cm)
k
Clonal tree
CTNNB1
COL11A1
IL6ST
IL6ST
Clonal structure of SH12
T01
T02
T03
T04
T05

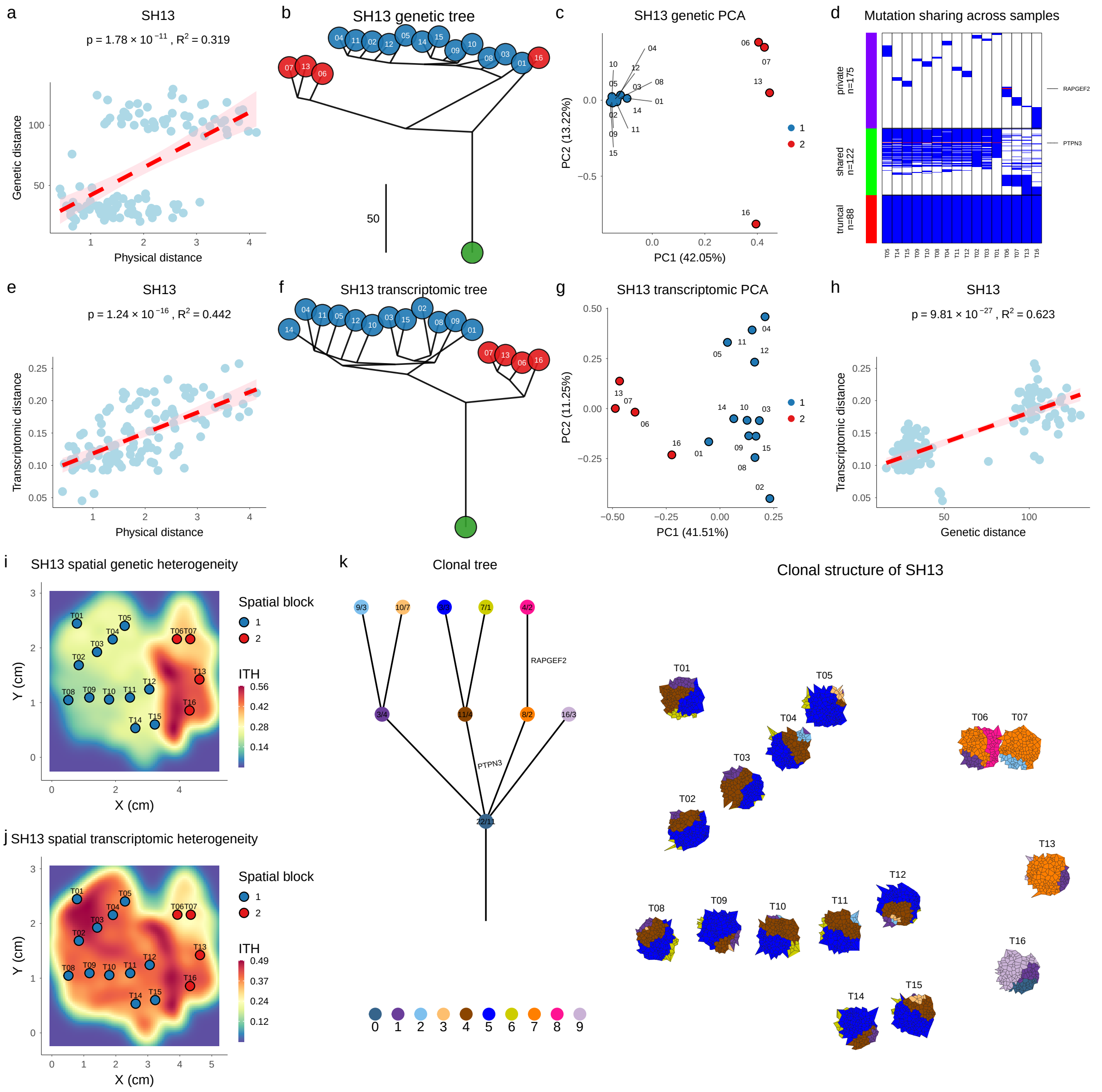

Supplementary Figure 3: The genomic characterization of the patient cohort. a) The regression between genetic differentiation (FST) and physical distance. b) The maximum parsimony tree built based on the genetic changes. c) PCA map constructed based on the genetic changes. Sectors were colored according to their spatial blocks. d) The present/absence of the mutations across sectors with truncal (red), branch (green) and tip (blue) labelled for each mutation (row). e) The regression between transcriptomic distance and physical distance. f) The phylogenetic relationship built based on the transcriptomic distances. g) PCA map constructed based on the transcriptomic profile. Sectors were colored according to their spatial blocks. h) The linear regression between genetic differences (FST) and transcriptomic distance. i)The spatial heatmap of the genetic spatial heterogeneity (the same figure as Figure 2e maintext). Sectors in different spatial blocks were labelled with different colors and were delineated using black margins. j) The spatial heatmap of the transcriptomic spatial heterogeneity. Sectors in different spatial blocks were labelled with different colors. k) The clonal composition of the tumor sectors. The clonal relationship and clonal composition of different sectors are shown. Driver mutations as well as the number of nonsynonymous/synonymous mutations in different clones were labelled on the branches as well as nodes. The corresponding plot for each patient (SH01-SH13) was shown on page 3-15. Source data are provided as a Source Data file.

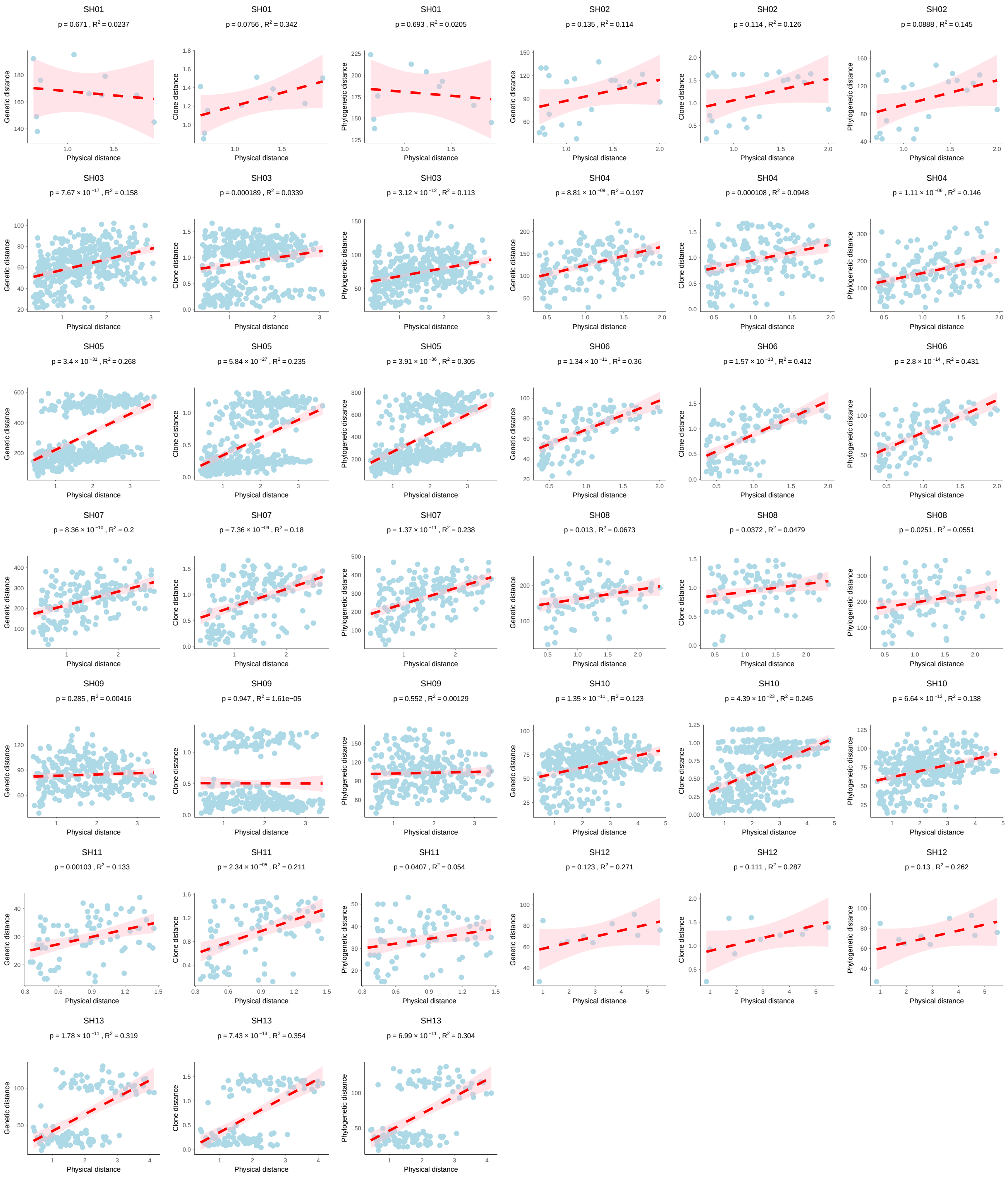

Supplementary Figure 4. The correlation between multiple metrics measuring genetic differentiation and physical distance. The correlation between physical distance and multiple metrics measuring genetic differentiation (FST, clonal distance as well as phylogenetic distance, see Methods) were plotted for the whole cohort. Source data are provided as a Source Data file.

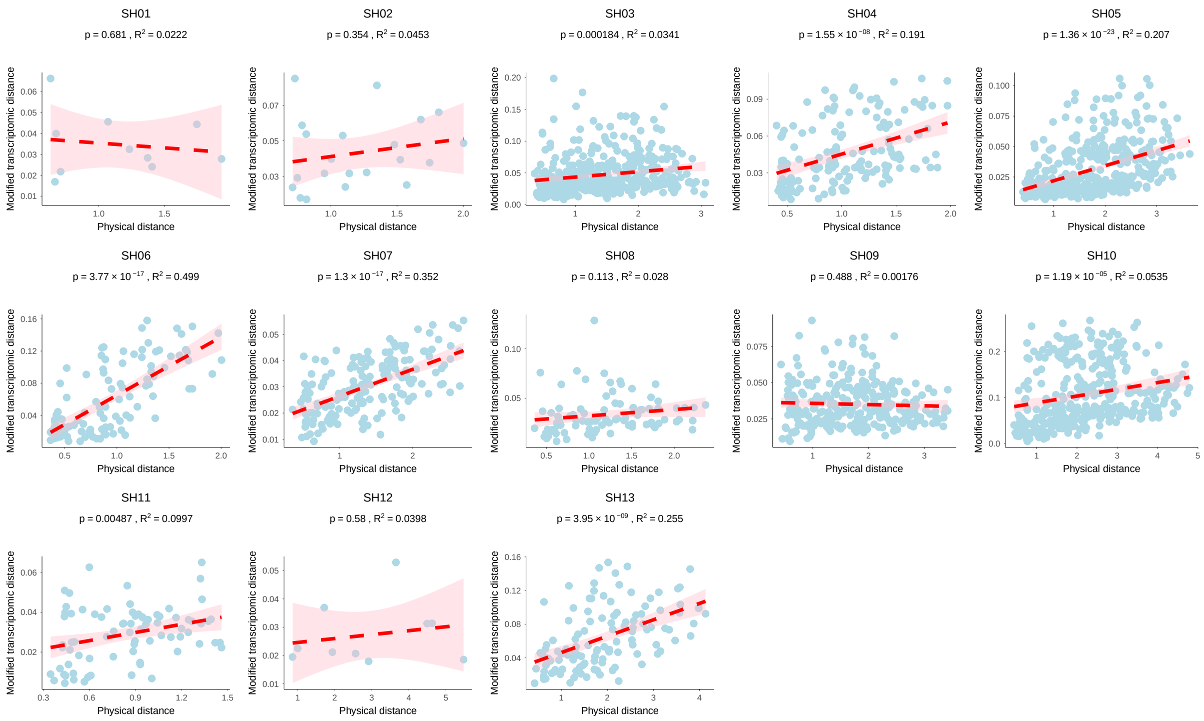

Supplementary Figure 5. The correlation between modified transcriptomic difference and physical distance. Transcriptomic distances calculated based on genes that are positively correlated with tumor purity (methods) were plotted against the physical distances between sectors for each patient. Source data are provided as a Source Data file.

Supplementary Figure 6: Multiple regression between transcriptomic distance and physical distance. (page 18-30 , see figure legend on page 30).

a

Multiple regression between transcriptomic distance and physical distance for SH01

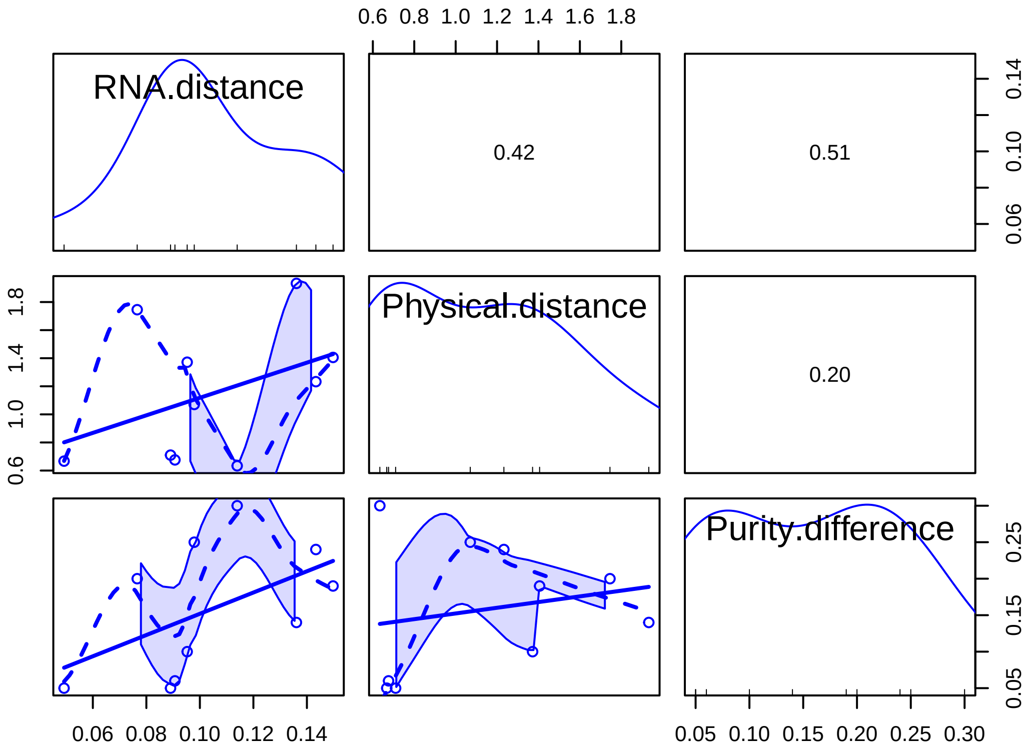

b

	Estimate	Standard Error	t value	Pr(>|t|)	
(Intercept)	0.055	0.028	1.976	0.0887	.
\`Physical distance\`	0.022	0.021	1.070	0.3203	
\`Purity difference\`	0.153	0.107	1.423	0.1976	

*Signif. codes: 0 <= '***' < 0.001 < '**' < 0.01 < '*' < 0.05*

Residual standard error: 0.02873 on 7 degrees of freedom

Multiple R-squared: 0.3606, Adjusted R-squared: 0.178

F-statistic: 1.974 on 7 and 2 DF, p-value: 0.2090

a

Multiple regression between transcriptomic distance and physical distance for SH02

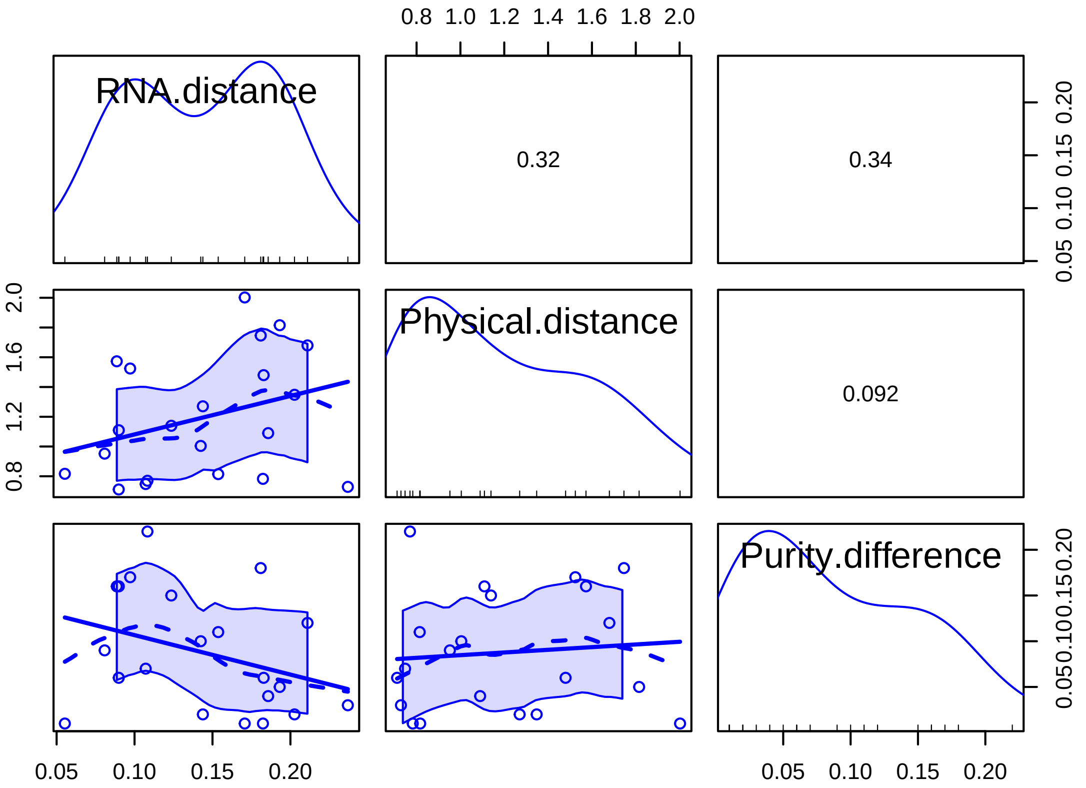

b

	Estimate	Standard Error	t value	Pr(>|t|)	
(Intercept)	0.116	0.034	3.405	0.0032	**
\`Physical distance\`	0.044	0.026	1.724	0.1018	
\`Purity difference\`	-0.289	0.161	-1.791	0.0901	.

*Signif. codes: 0 <= '***' < 0.001 < '**' < 0.01 < '*' < 0.05*

Residual standard error: 0.04682 on 18 degrees of freedom

Multiple R-squared: 0.2393, Adjusted R-squared: 0.1548

F-statistic: 2.832 on 18 and 2 DF, p-value: 0.0853

a

Multiple regression between transcriptomic distance and physical distance for SH03

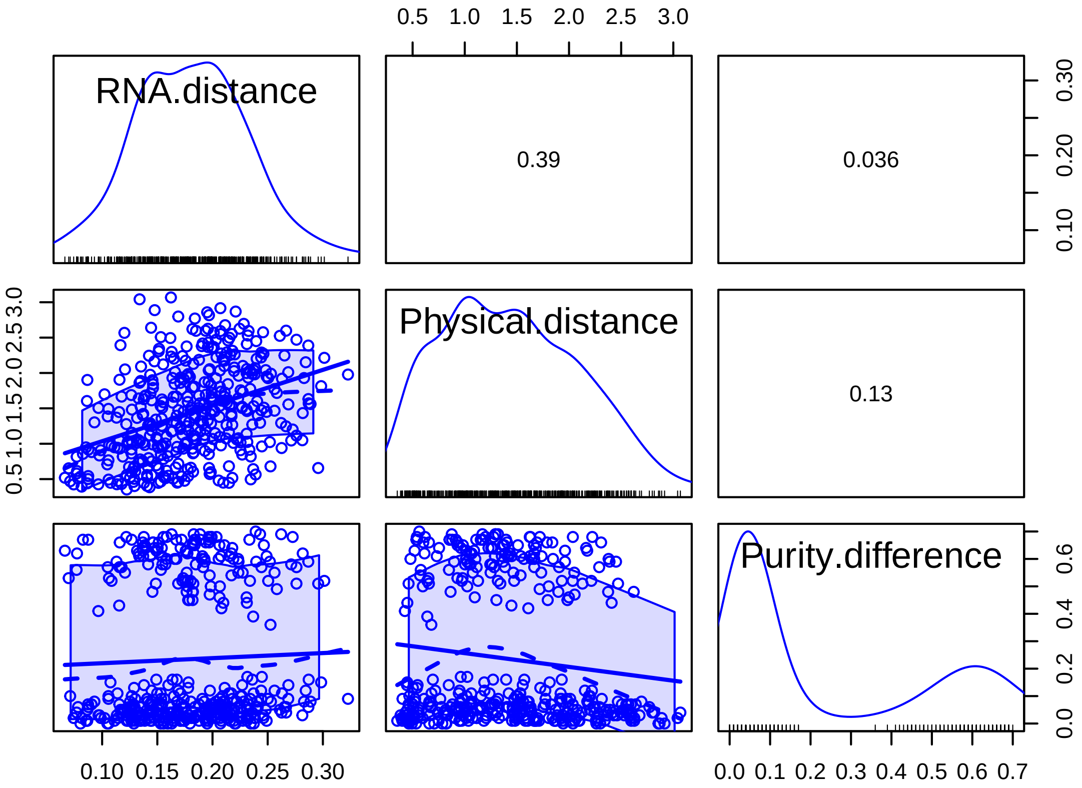

b

	Estimate	Standard Error	t value	Pr(>|t|)	
(Intercept)	0.132	0.006	21.321	0.0000	***
\`Physical distance\`	0.031	0.004	8.642	0.0000	***
\`Purity difference\`	0.017	0.009	1.870	0.0622	.

*Signif. codes: 0 <= '***' < 0.001 < '**' < 0.01 < '*' < 0.05*

Residual standard error: 0.0462 on 403 degrees of freedom

Multiple R-squared: 0.1574, Adjusted R-squared: 0.1532

F-statistic: 37.65 on 403 and 2 DF, p-value: 0.0000

a

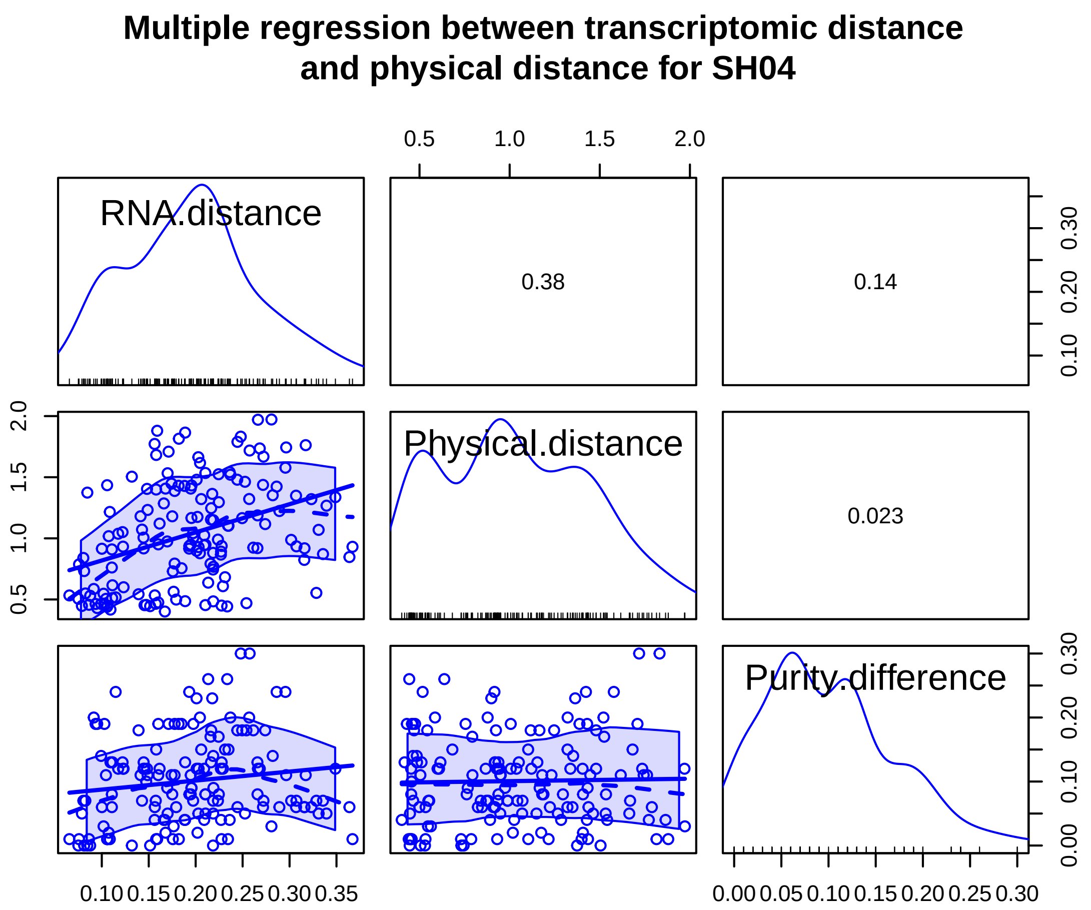

b

	Estimate	Standard Error	t value	Pr(>|t|)	
(Intercept)	0.116	0.016	7.360	0.0000	***
`Physical distance`	0.063	0.012	5.082	0.0000	***
`Purity difference`	0.135	0.076	1.790	0.0755	.

*Signif. codes: 0 <= '***' < 0.001 < '**' < 0.01 < '*' < 0.05*

Residual standard error: 0.06503 on 150 degrees of freedom

Multiple R-squared: 0.1642, Adjusted R-squared: 0.153

F-statistic: 14.73 on 150 and 2 DF, p-value: 0.0000

a

Multiple regression between transcriptomic distance and physical distance for SH05

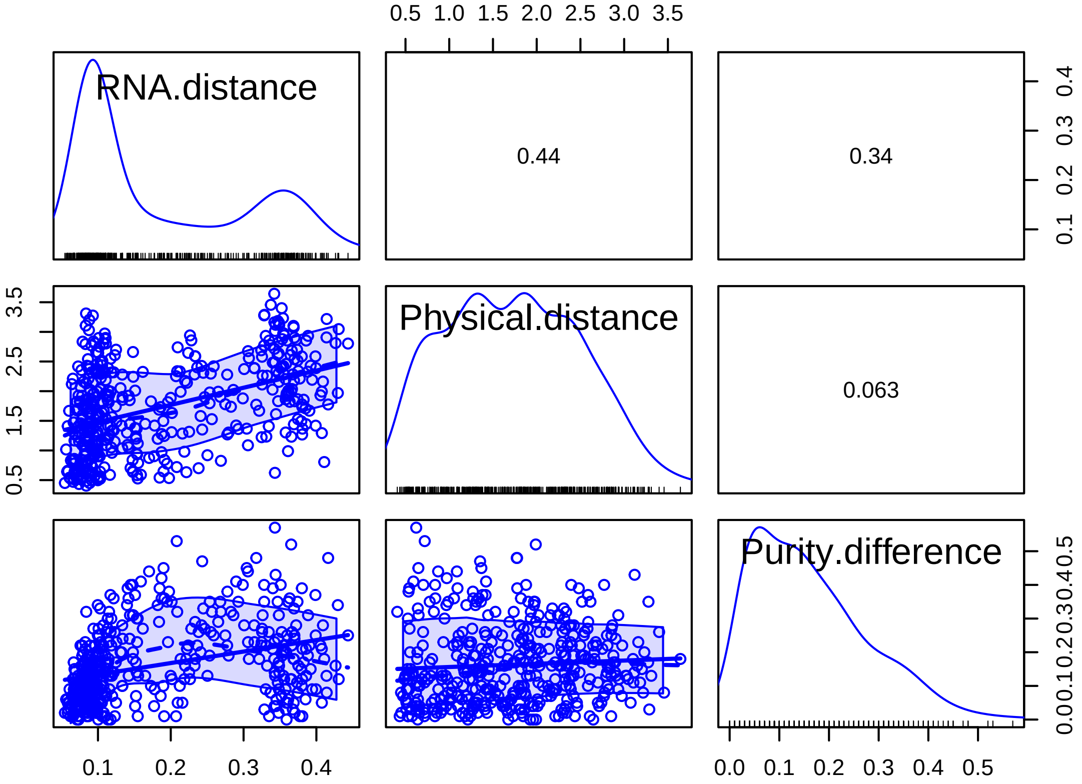

b

	Estimate	Standard Error	t value	Pr(>|t|)	
(Intercept)	0.023	0.013	1.723	0.0856	.
`Physical distance`	0.065	0.006	10.472	0.0000	***
`Purity difference`	0.316	0.040	7.799	0.0000	***

*Signif. codes: 0 <= '***' < 0.001 < '**' < 0.01 < '*' < 0.05*

Residual standard error: 0.09847 on 432 degrees of freedom

Multiple R-squared: 0.2958, Adjusted R-squared: 0.2925

F-statistic: 90.73 on 432 and 2 DF, p-value: 0.0000

a

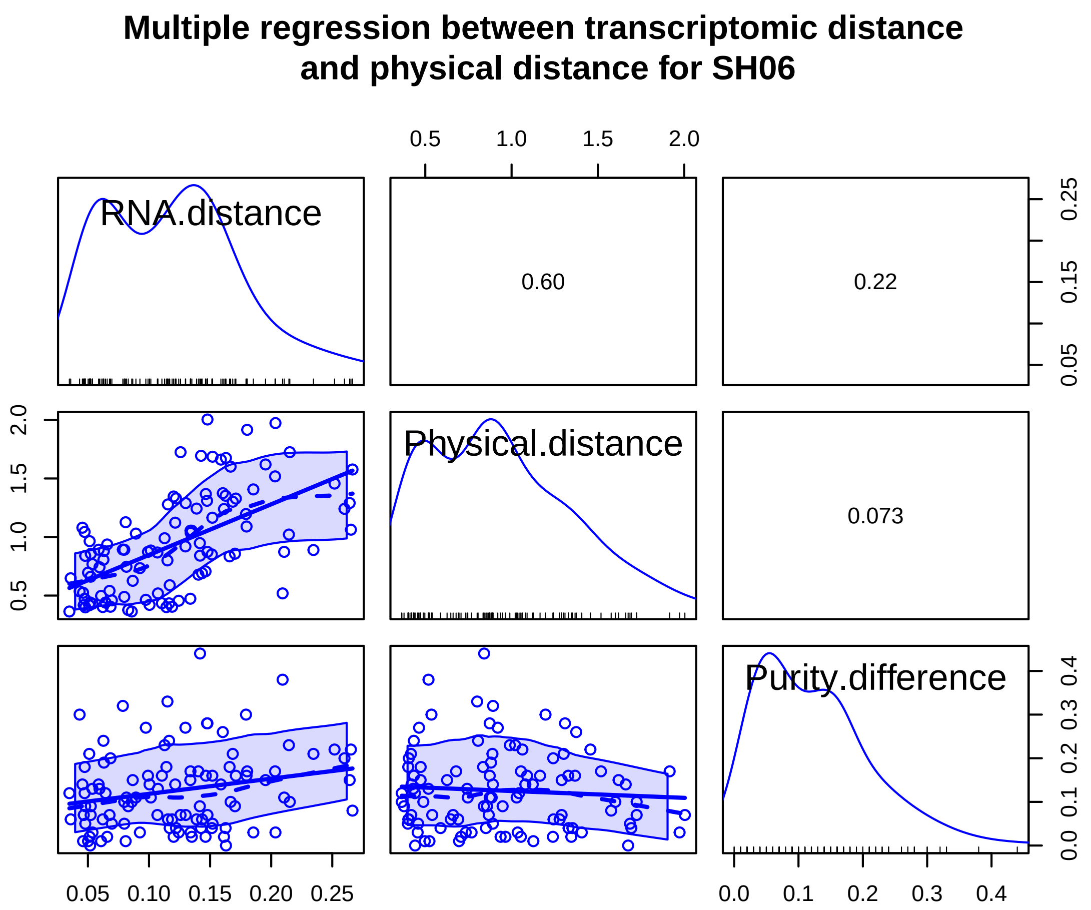

b

	Estimate	Standard Error	t value	Pr(>|t|)	
(Intercept)	0.019	0.013	1.533	0.1285	
\`Physical distance\`	0.085	0.010	8.244	0.0000	***
\`Purity difference\`	0.173	0.048	3.589	0.0005	***

*Signif. codes: 0 <= '***' < 0.001 < '**' < 0.01 < '*' < 0.05*

Residual standard error: 0.04472 on 102 degrees of freedom

Multiple R-squared: 0.43, Adjusted R-squared: 0.4188

F-statistic: 38.47 on 102 and 2 DF, p-value: 0.0000

a

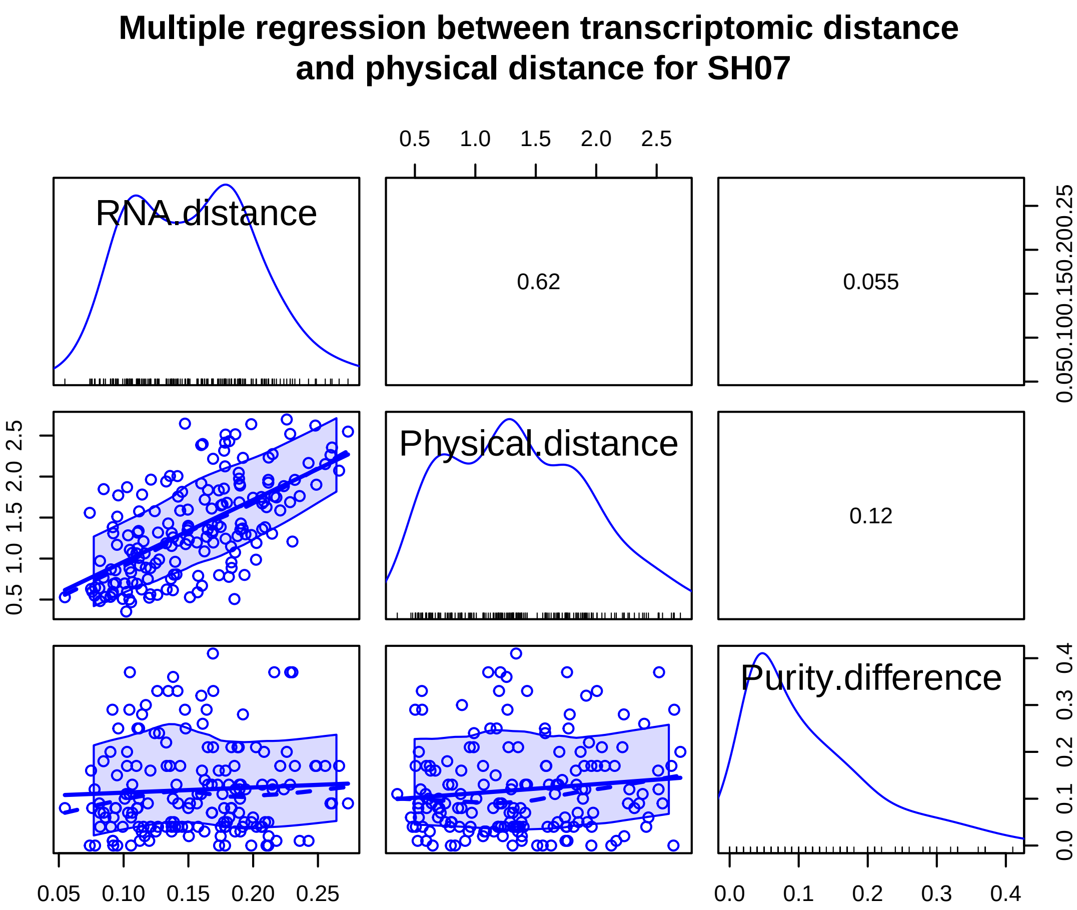

b

	Estimate	Standard Error	t value	Pr(>|t|)	
(Intercept)	0.085	0.008	10.788	0.0000	***
\`Physical distance\`	0.051	0.005	10.243	0.0000	***
\`Purity difference\`	-0.009	0.030	-0.292	0.7707	

*Signif. codes: 0 <= '***' < 0.001 < '**' < 0.01 < '*' < 0.05*

Residual standard error: 0.03823 on 168 degrees of freedom

Multiple R-squared: 0.3863, Adjusted R-squared: 0.379

F-statistic: 52.88 on 168 and 2 DF, p-value: 0.0000

a

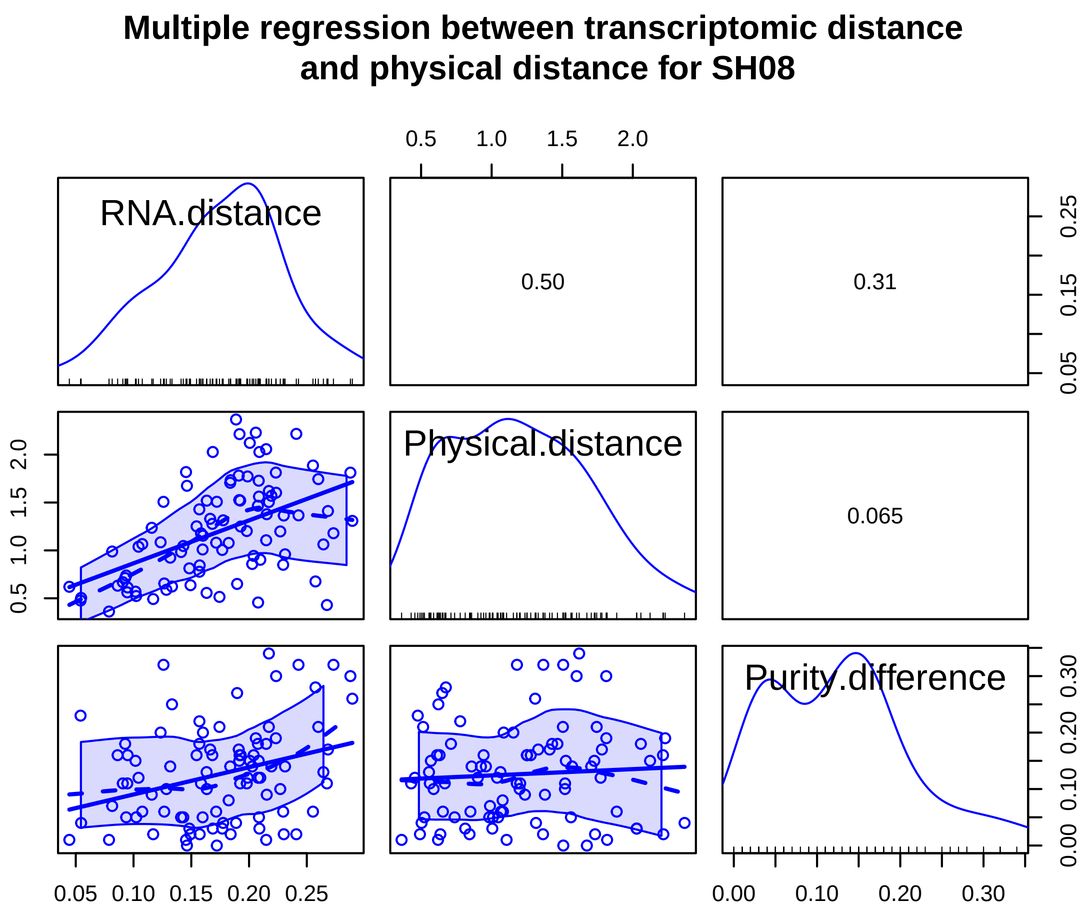

b

	Estimate	Standard Error	t value	Pr(>|t|)	
(Intercept)	0.087	0.014	6.163	0.0000	***
`Physical distance`	0.053	0.010	5.468	0.0000	***
`Purity difference`	0.185	0.057	3.235	0.0017	**

*Signif. codes: 0 <= '***' < 0.001 < '**' < 0.01 < '*' < 0.05*

Residual standard error: 0.04654 on 88 degrees of freedom

Multiple R-squared: 0.3275, Adjusted R-squared: 0.3122

F-statistic: 21.43 on 88 and 2 DF, p-value: 0.0000

a

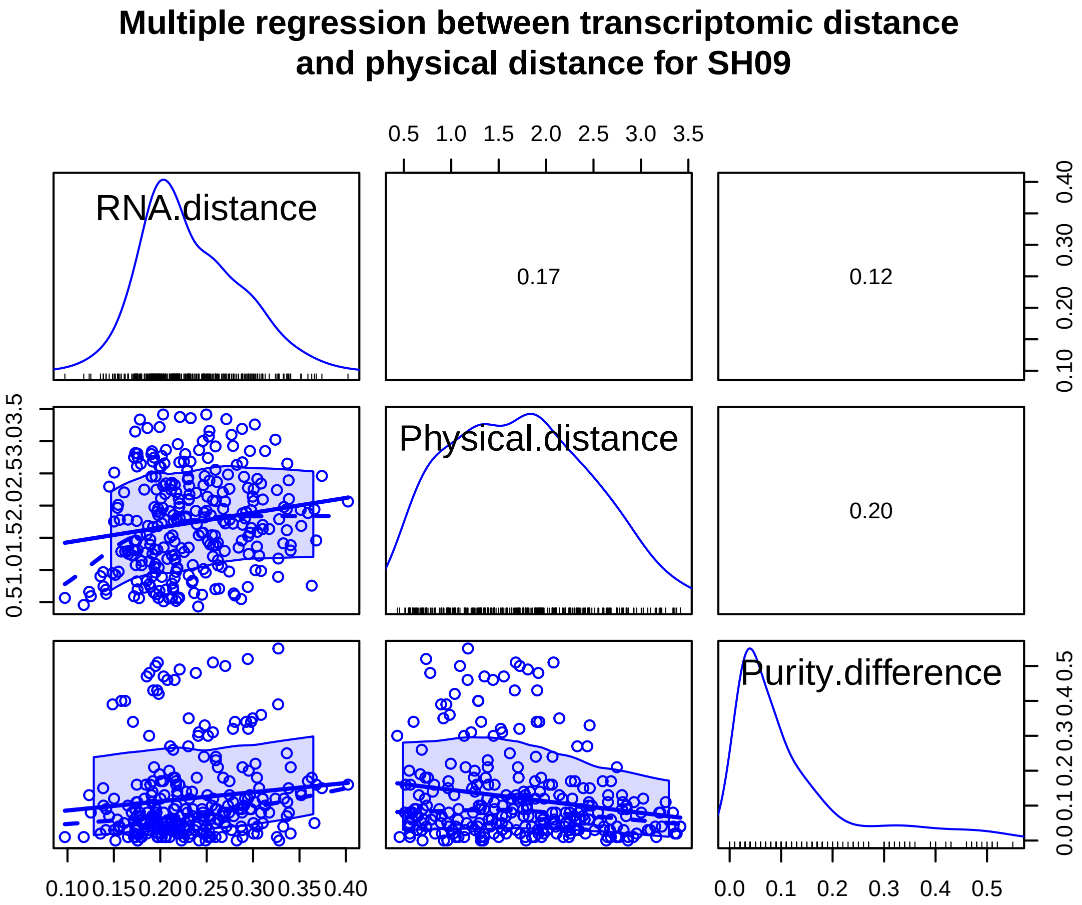

b

	Estimate	Standard Error	t value	Pr(>|t|)	
(Intercept)	0.199	0.009	21.120	0.0000	***
`Physical distance`	0.015	0.004	3.317	0.0010	**
`Purity difference`	0.068	0.026	2.573	0.0106	*

*Signif. codes: 0 <= '***' < 0.001 < '**' < 0.01 < '*' < 0.05*

Residual standard error: 0.05411 on 273 degrees of freedom

Multiple R-squared: 0.05153, Adjusted R-squared: 0.04458

F-statistic: 7.417 on 273 and 2 DF, p-value: 0.0007

a

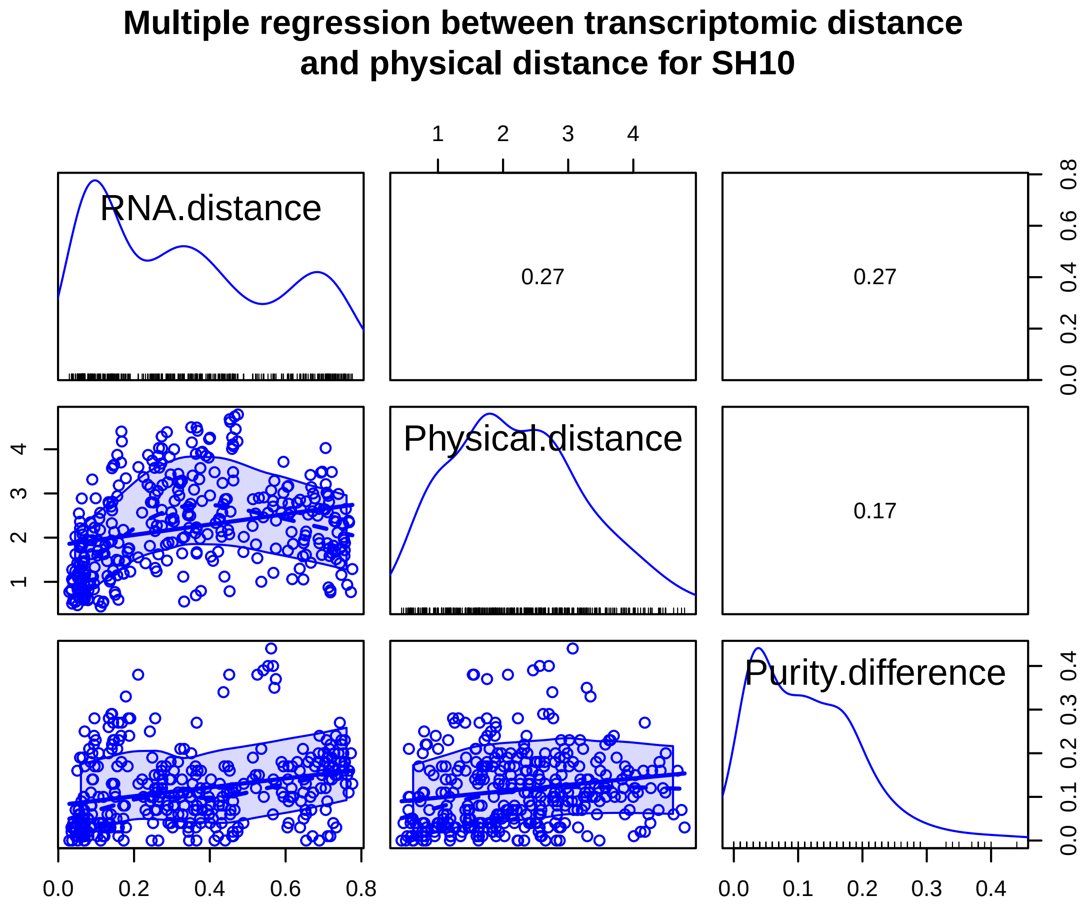

b

	Estimate	Standard Error	t value	Pr(>|t|)	
(Intercept)	0.156	0.030	5.159	0.0000	***
\`Physical distance\`	0.052	0.012	4.466	0.0000	***
\`Purity difference\`	0.632	0.137	4.622	0.0000	***

*Signif. codes: 0 <= '***' < 0.001 < '**' < 0.01 < '*' < 0.05*

Residual standard error: 0.2206 on 348 degrees of freedom

Multiple R-squared: 0.1255, Adjusted R-squared: 0.1205

F-statistic: 24.98 on 348 and 2 DF, p-value: 0.0000

a

Multiple regression between transcriptomic distance and physical distance for SH11

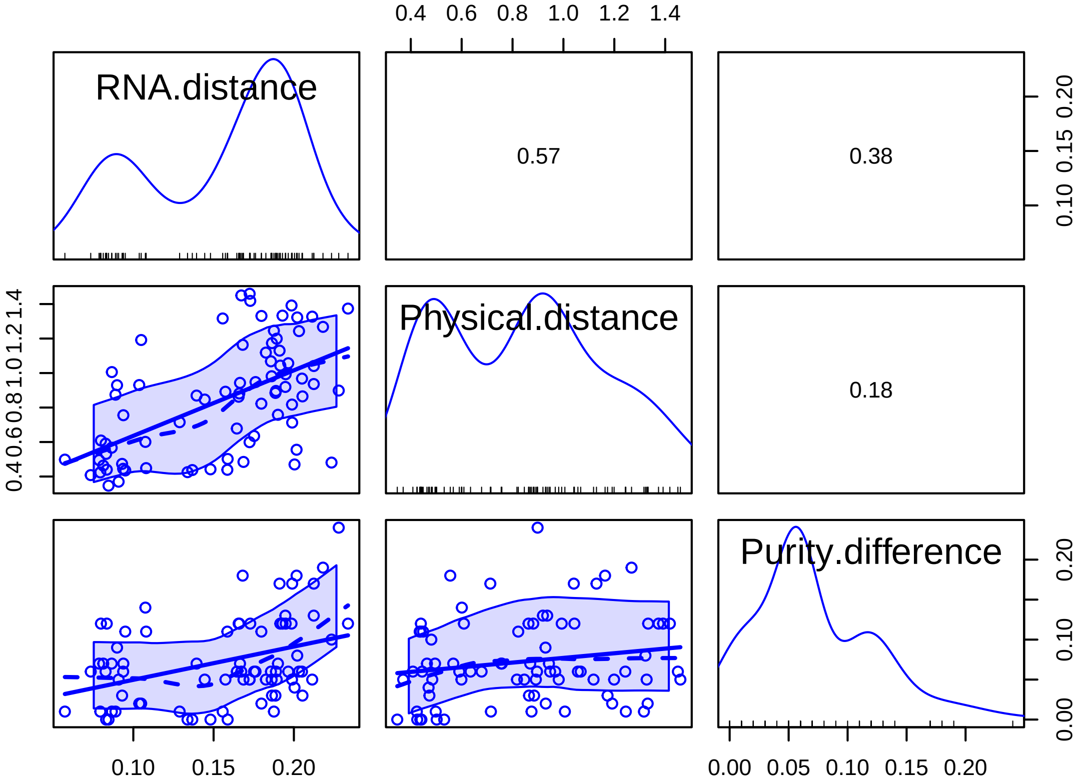

b

	Estimate	Standard Error	t value	Pr(>|t|)	
(Intercept)	0.071	0.013	5.590	0.0000	***
`Physical distance`	0.077	0.014	5.685	0.0000	***
`Purity difference`	0.261	0.083	3.161	0.0023	**

*Signif. codes: 0 <= '***' < 0.001 < '**' < 0.01 < '*' < 0.05*

Residual standard error: 0.03787 on 75 degrees of freedom

Multiple R-squared: 0.4014, Adjusted R-squared: 0.3855

F-statistic: 25.15 on 75 and 2 DF, p-value: 0.0000

a

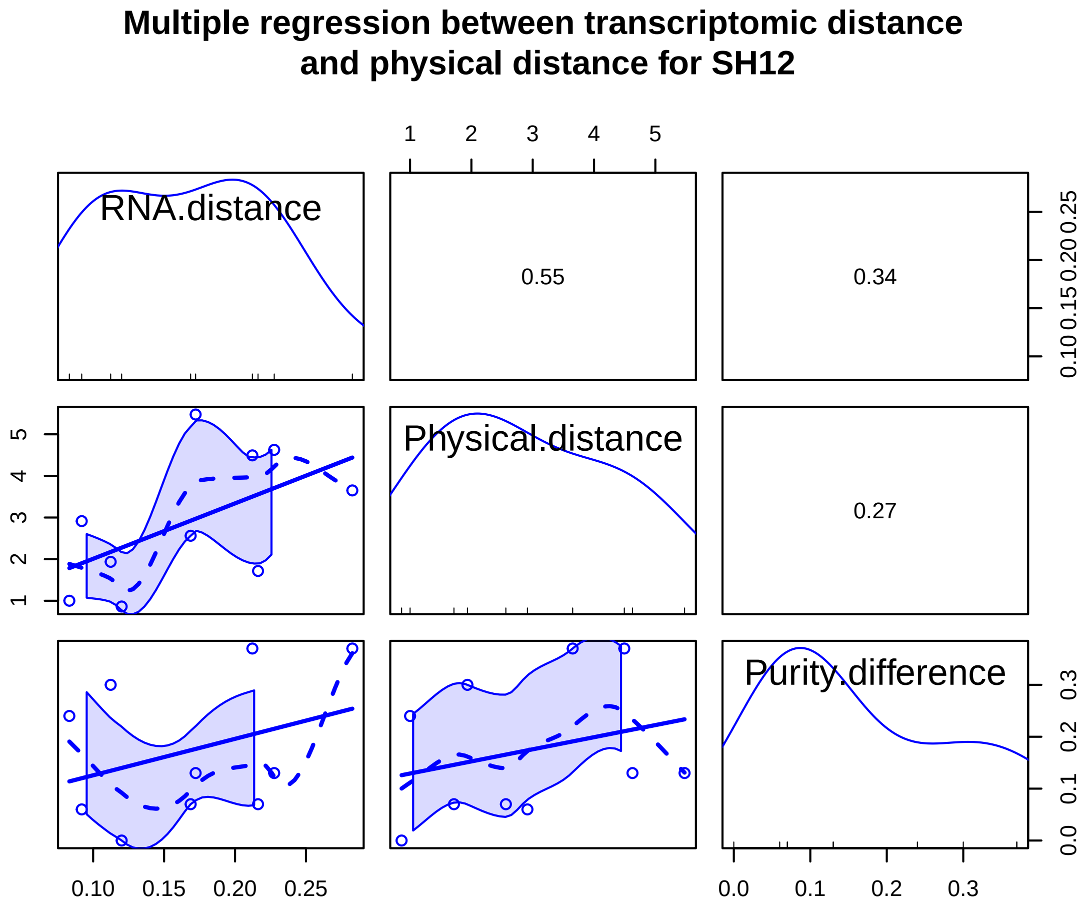

b

	Estimate	Standard Error	t value	Pr(>|t|)	
(Intercept)	0.091	0.045	2.034	0.0815	.
\`Physical distance\`	0.021	0.013	1.556	0.1637	
\`Purity difference\`	0.101	0.155	0.648	0.5375	

*Signif. codes: 0 <= '***' < 0.001 < '**' < 0.01 < '*' < 0.05*

Residual standard error: 0.06073 on 7 degrees of freedom

Multiple R-squared: 0.344, Adjusted R-squared: 0.1565

F-statistic: 1.835 on 7 and 2 DF, p-value: 0.2287

a

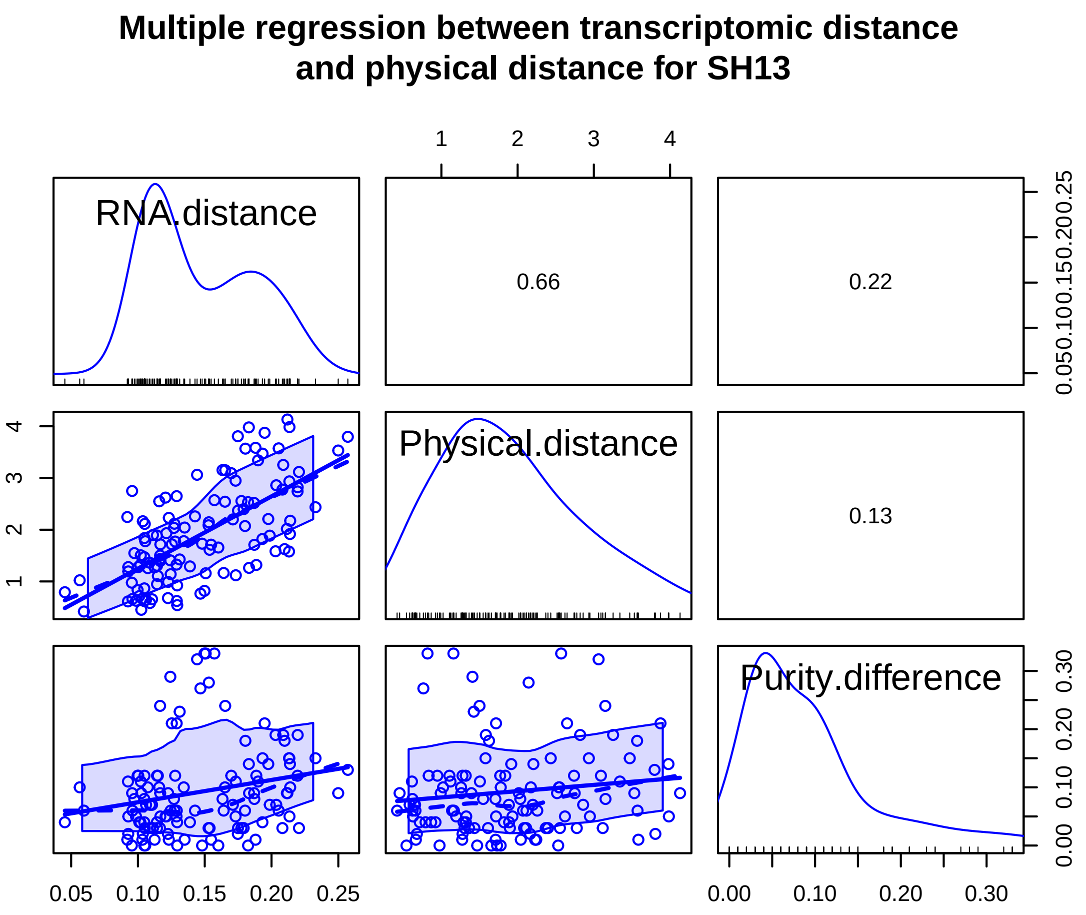

b

<table>
<tr><th></th><th>Estimate</th><th>Standard Error</th><th>t value</th><th>Pr(>|t|)</th><th></th></tr>
<tr><td>(Intercept)</td><td>0.081</td><td>0.007</td><td>10.958</td><td>0.0000</td><td>***</td></tr>
<tr><td>`Physical distance`</td><td>0.031</td><td>0.003</td><td>9.448</td><td>0.0000</td><td>***</td></tr>
<tr><td>`Purity difference`</td><td>0.077</td><td>0.039</td><td>1.962</td><td>0.0521</td><td>.</td></tr>
</table>

Signif. codes: 0 <= '***' < 0.001 < '**' < 0.01 < '*' < 0.05

Residual standard error: 0.03304 on 117 degrees of freedom

Multiple R-squared: 0.4597, Adjusted R-squared: 0.4504

F-statistic: 49.77 on 117 and 2 DF, p-value: 0.0000

Supplementary Figure 6. Multiple regression between transcriptomic distance and physical distance. a) The pairwise regression plot between transcriptomic distance (RNA distance), physical distance and tumor purity differences. b) The statistical output of the multiple regression RNA_distance ∼ Physical_distance + Purity_difference. The corresponding plot for each patient (SH01-SH13) was shown on page 18-30. Source data are provided as a Source Data file.

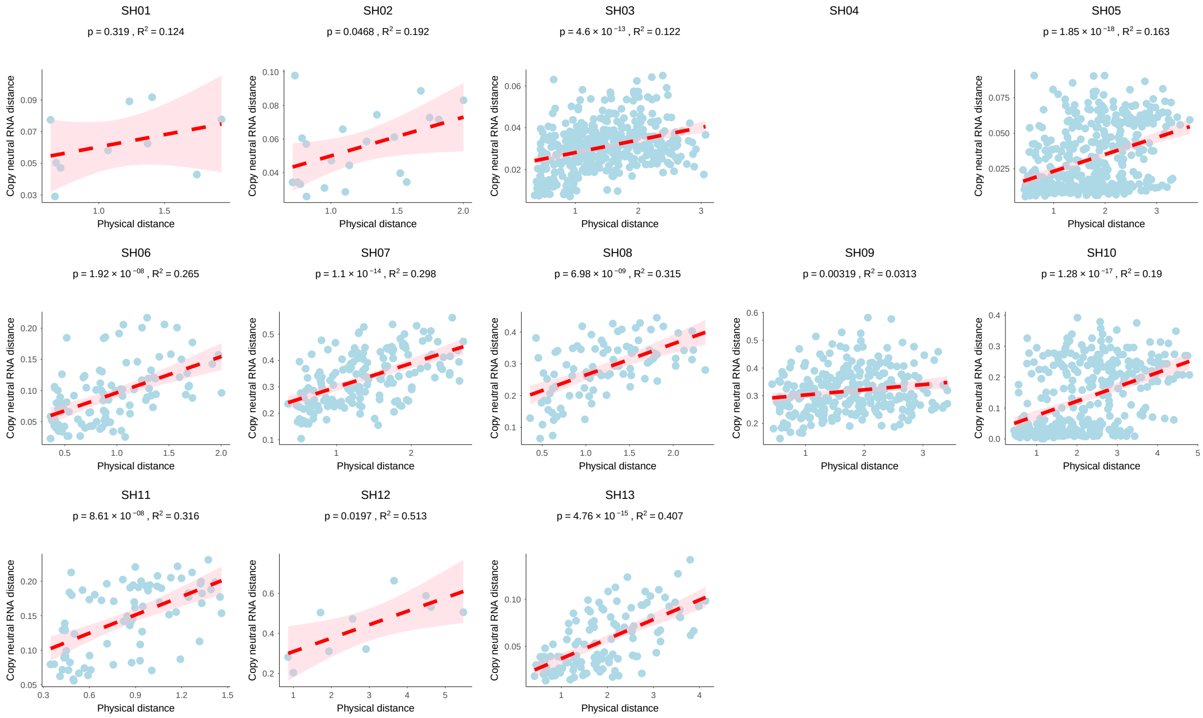

Supplementary Figure 7. The correlation between transcriptomic difference based on copy neutral regions and physical distance. Transcriptomic distances calculated based on genes from copy neutral regions (methods) were plotted against the physical distances between sectors. SH04 has no copy neutral regions and was not plotted for this analysis. Source data are provided as a Source Data file.

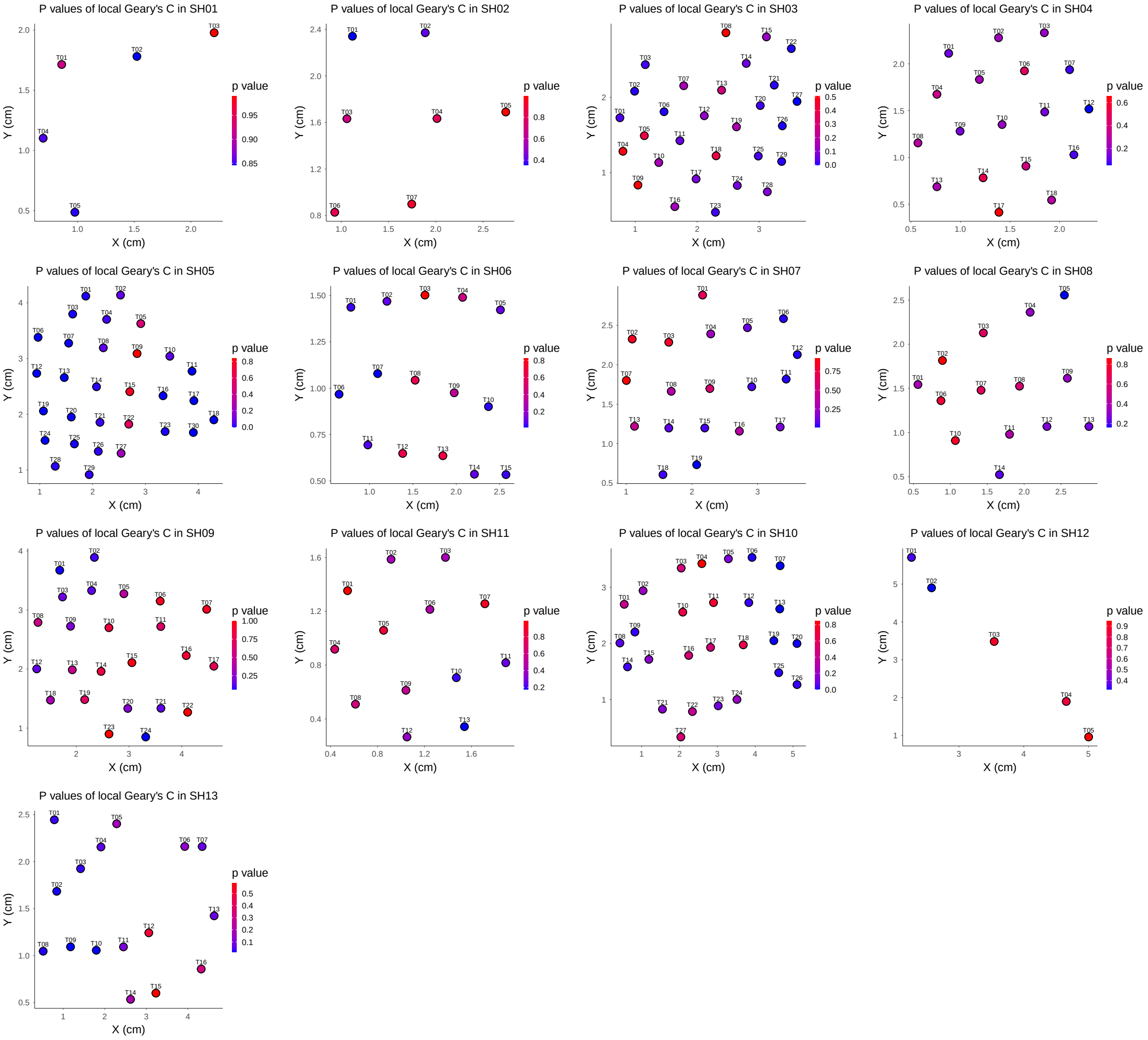

Supplementary Figure 8. Geary's C statistics and its significance. For each sector, we first calculated Geary's C value (Methods) and subsequently test the significance of the observed Geary's C value by randomly shuffling the sectors across spatial locations of the sectors (Methods). We then plotted the p-values across the sectors and found that sectors at the boundary of the spatial blocks tend to have weak significance due to weak spatial correlation. Source data are provided as a Source Data file.

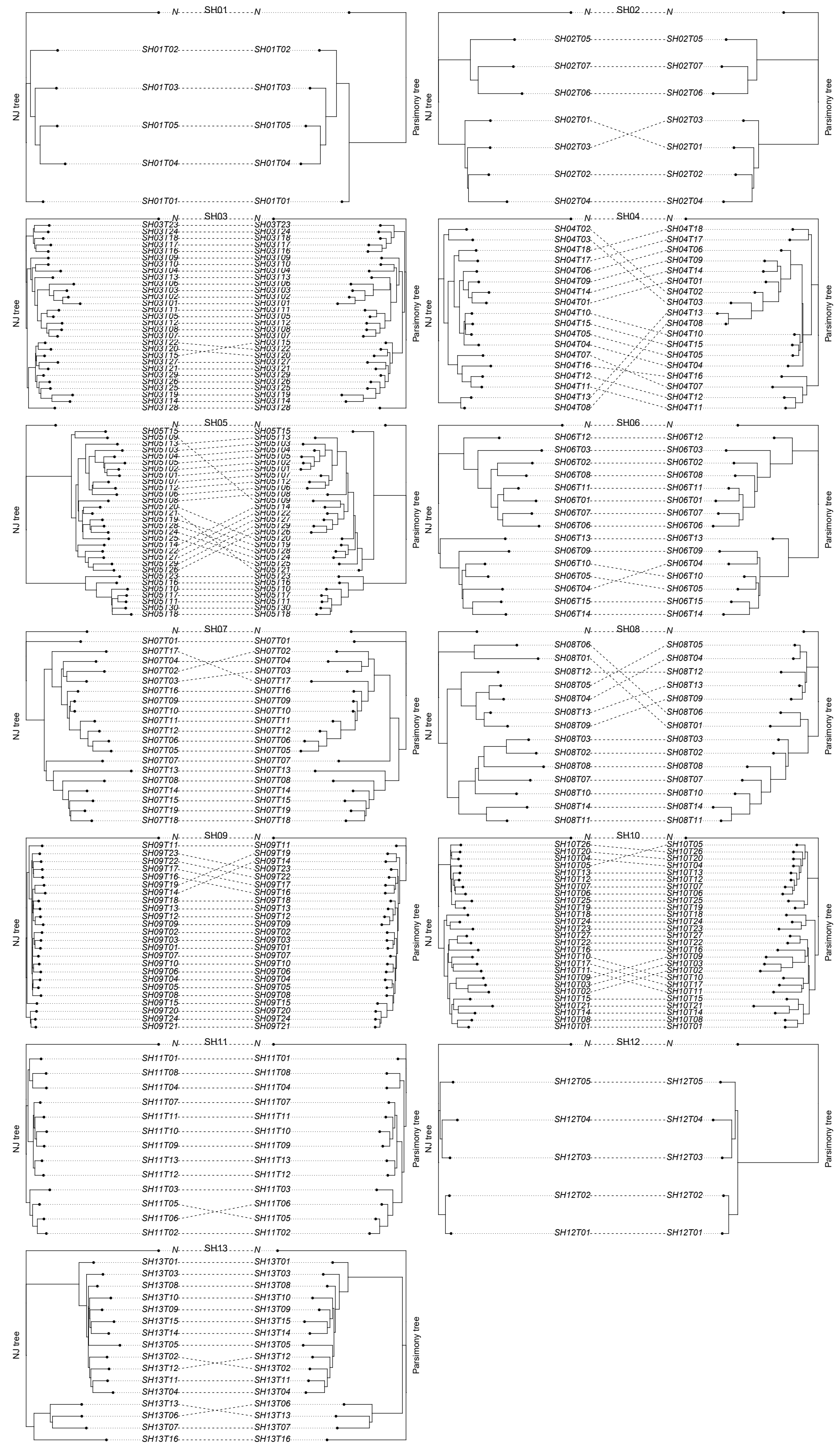

Supplementary Figure 9. Comparison of phylogenetic trees inferred using different methods. Phylogenetic trees inferred based on neighbor joining principle (left) were compared with trees inferred using maximum parsimony (right).

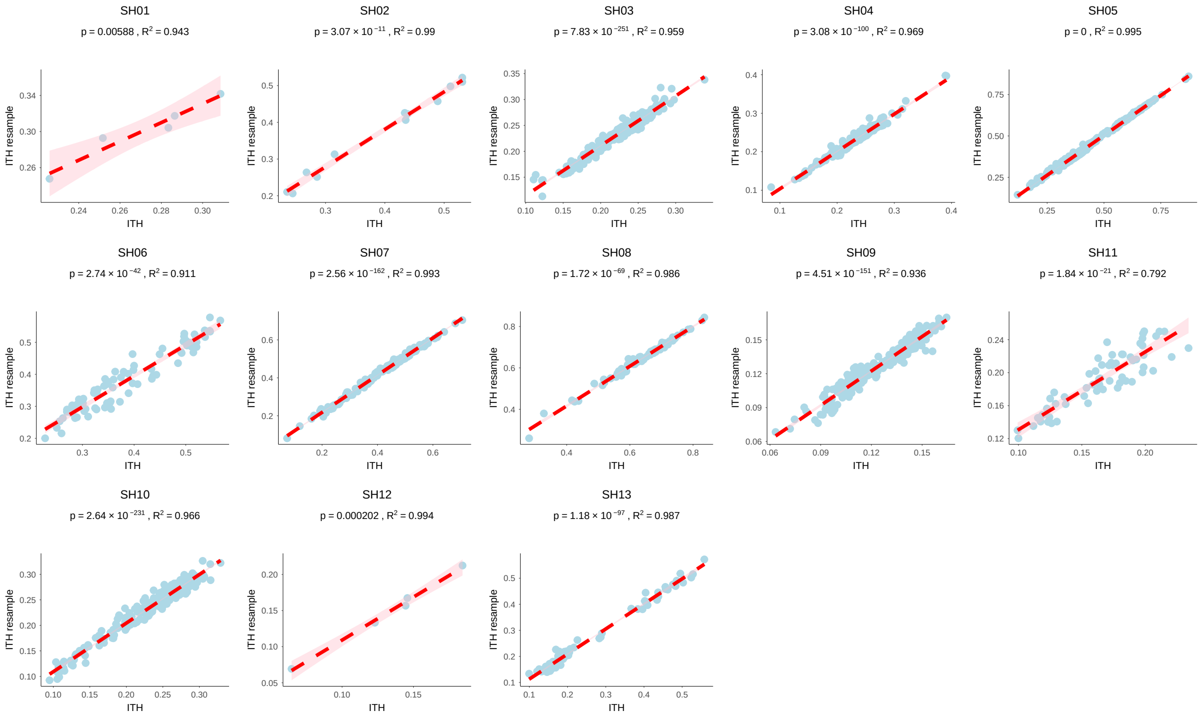

Supplementary Figure 10. Sequencing depth and θ_R. In order to examining the effect of sequencing depth on θ_R, we downsampled the sequencing depth of all sectors from a patient to the minimum depth across sectors. For each downsampled sector, we recalled mutations and recalculated θ_R. We found that the original θ_R and θ_R from downsampled datasets are highly similar. In other words, sequencing depth has very minor affect on θ_R.

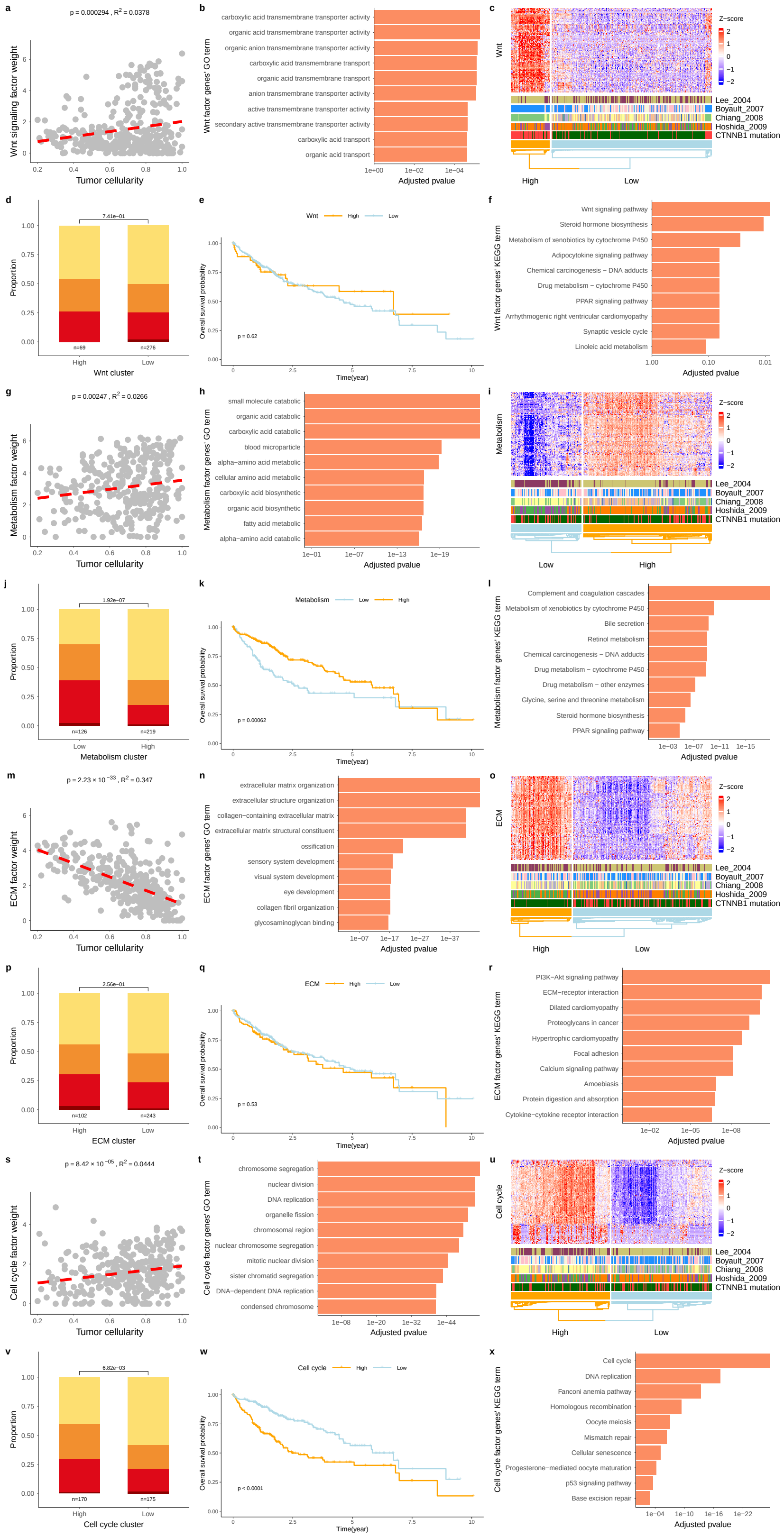

Supplementary Figure 11. The genomic characterization of the compartments. a) The regression between tumor cellularity (purity) and factor weight of the Wnt compartment. b) The GO enrichment output for factor genes from the Wnt compartment. c) Clustering of the LIHC cohort based on factor genes from the Wnt compartment. d) The clinical stage of the Wnt high and low subgroups. e) Survival plot for the Wnt high and low subgroups. f) The KEGG enrichment for factor genes from the Wnt compartment. g-l) the corresponding plots for the metabolism compartment. m-r) the corresponding plots for the ECM compartment. s-x) the corresponding plots for the cell cycle compartment. Source data are provided as a Source Data file.

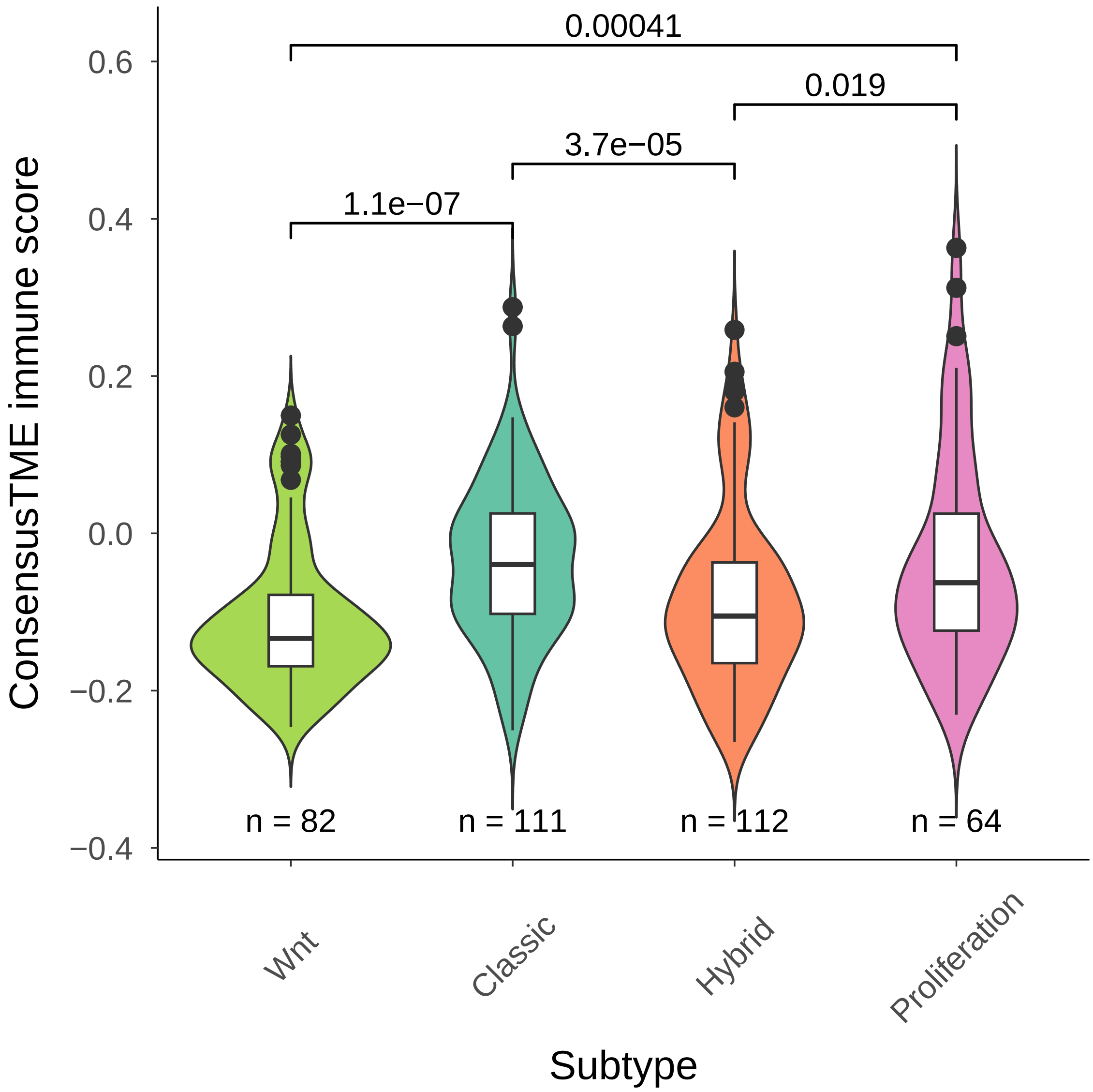

Supplementary Figure 12. The immune score across the transcriptomic subtypes. The boxplot of immune scores calculated based on the consensusTME for the LIHC cohort. The lower and upper hinges represent the 25th and 75th percentiles, respectively. Whiskers extend up to 1.5 * IQR from the hinges. Source data are provided as a Source Data file.

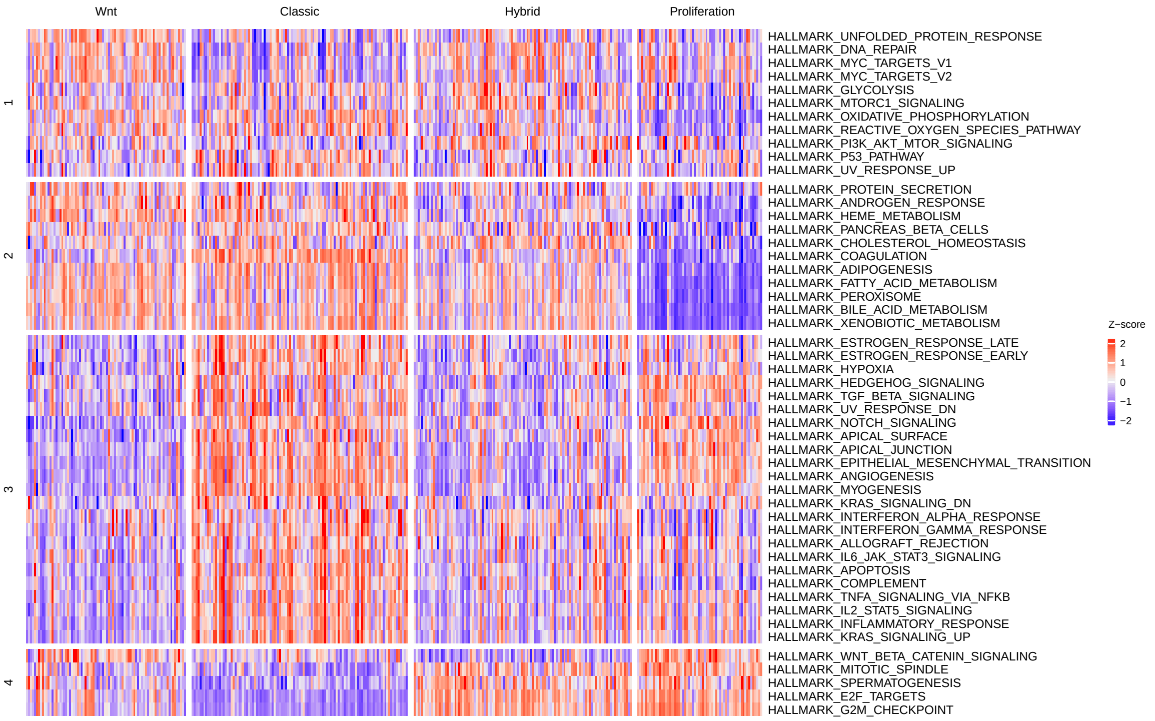

Supplementary Figure 13. GSVA scores of the HALLMARK pathways across the four subtypes. The value was normalized to Z-score across individuals (row).

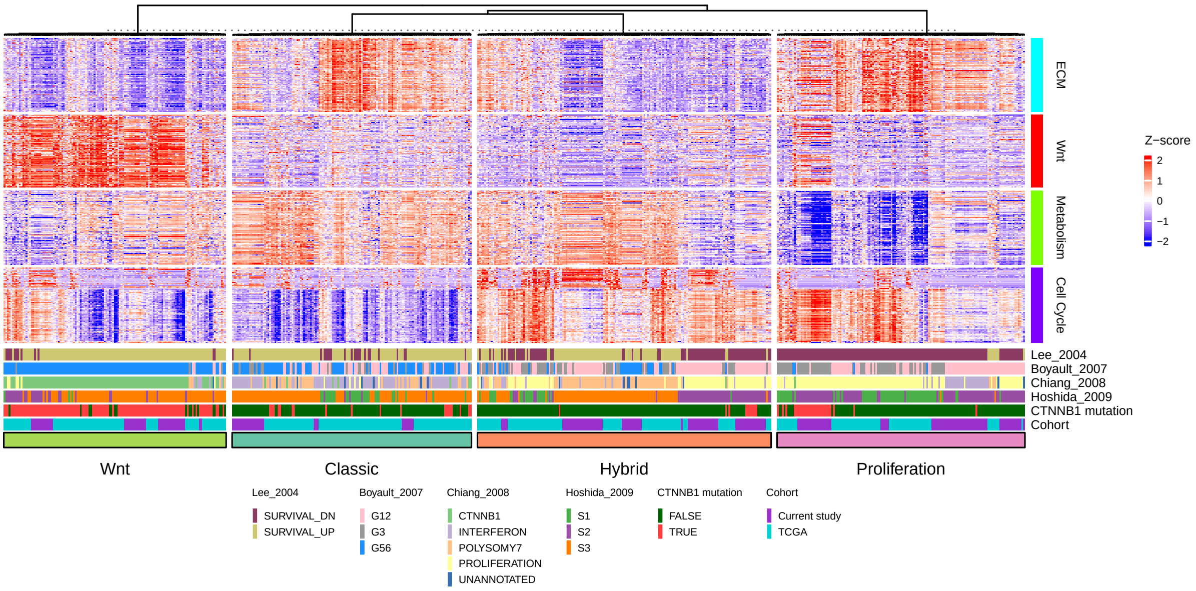

Supplementary Figure 14. Joint clustering of the TCGA and our own samples. The literature known subtypes, CTNNB1 mutations and the source (i.e. cohort) of samples were marked below the patients.

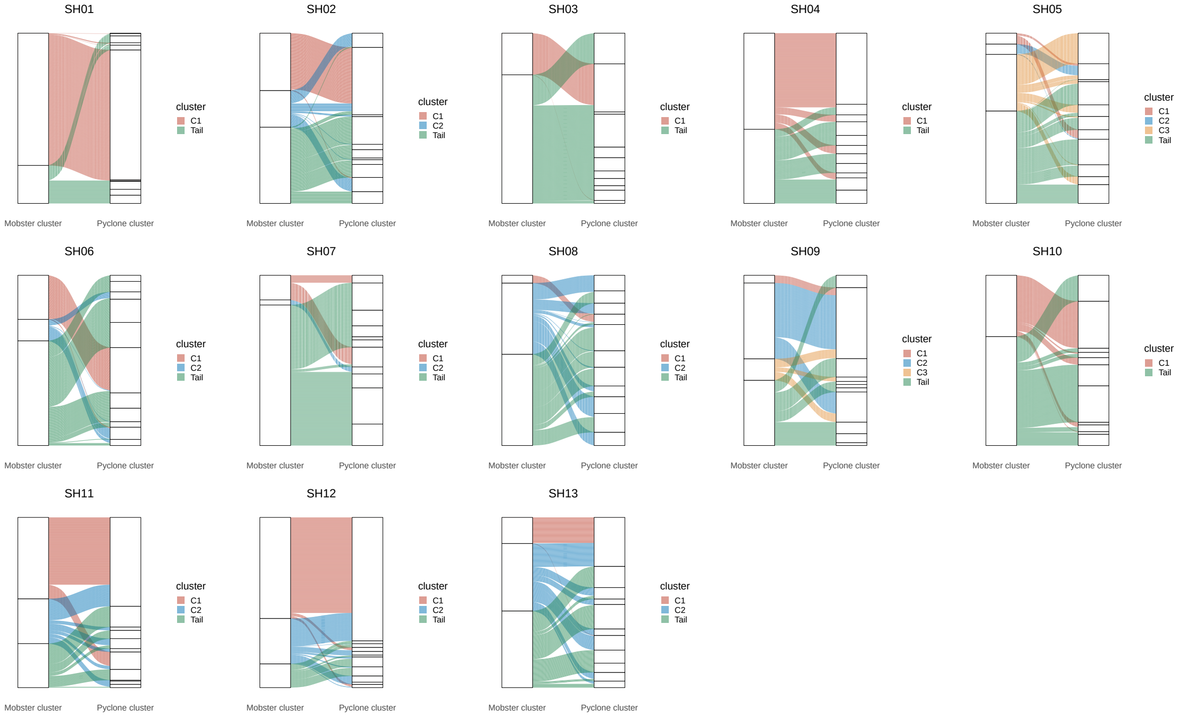

Supplementary Figure 15: Comparison of clonal structure between PyClone and MOBSTER. Mutations were combined across sectors to generate a single sample for clonal deconvolution using MOBSTER, while mutations across multiple sectors were input to PyClone for clonal deconvolution. This is an alluvial plot of the clustering of mutations between PyClone and MOBSTER. Each line is a mutation. The boxes on both ends mark the clones. For example, MOBSTER identifies both neutral tails (bottom box) and a clonal peak (upper box, C1). PyClone, on the other hand, identifies multiple subclones (many boxes, on the right). The clonal cluster identified by MOBSTER corresponds well to a major clone identified by PyClone. Because Pyclone models allele frequencies across multiple samples, it can further partition the neutral tails from a single sample into multiple subclones.

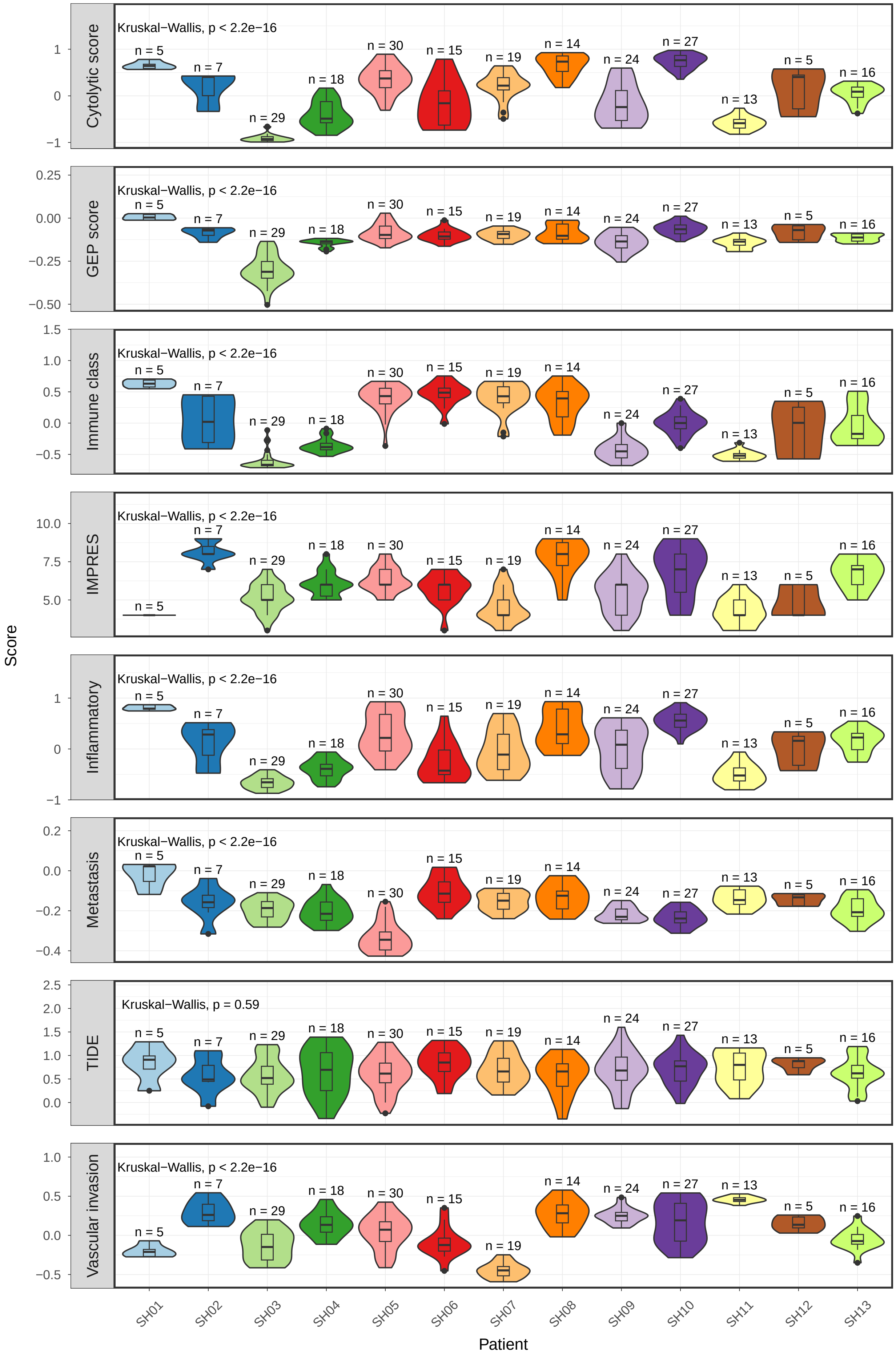

Supplementary Figure 16. Variation of the biomarkers across the patients. The boxplot of biomarkers across patients for the list of different biomarkers. The lower and upper hinges represent the 25th and 75th percentiles, respectively. Whiskers extend up to 1.5 * IQR from the hinges. Source data are provided as a Source Data file.

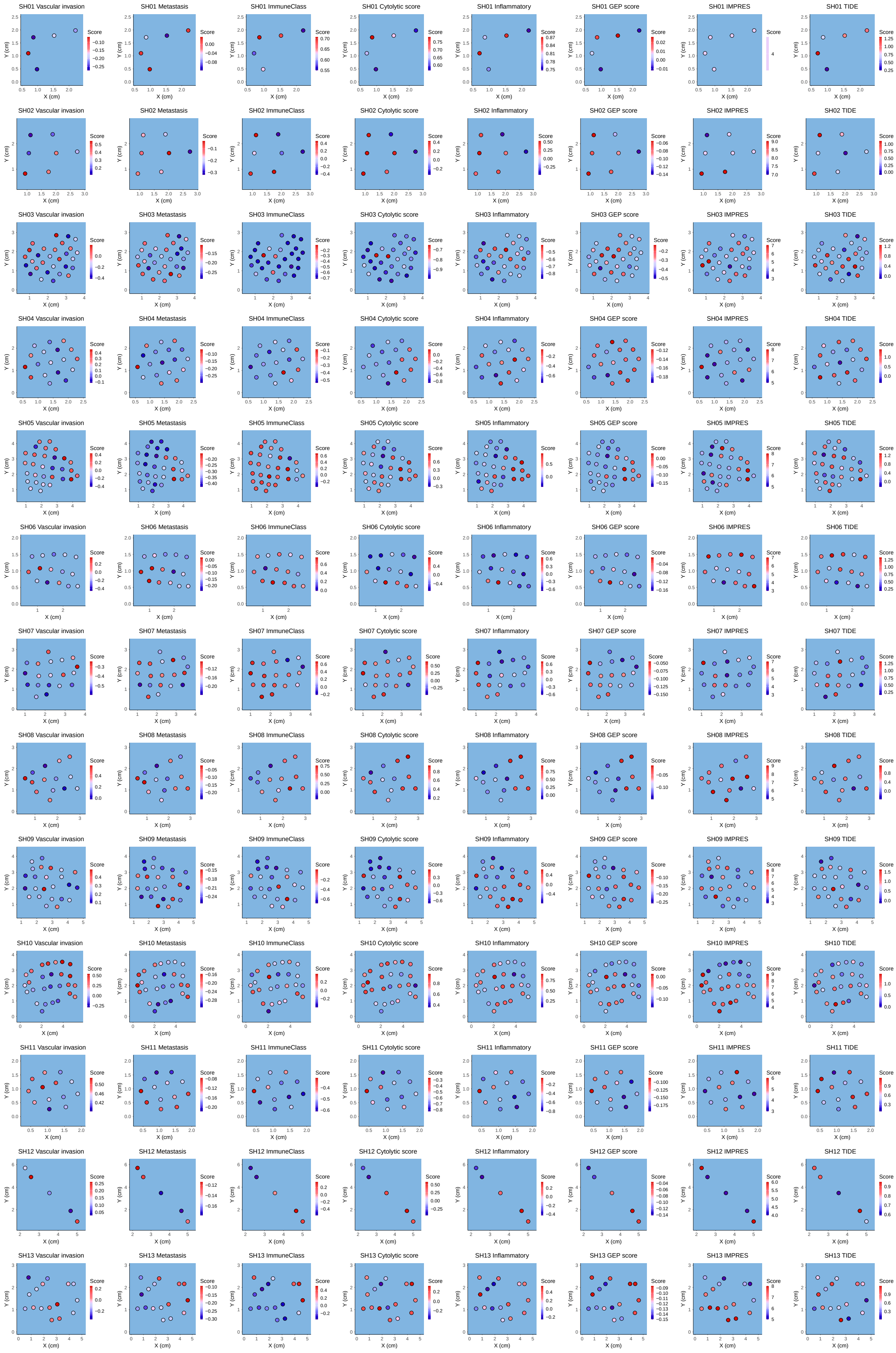

Supplementary Figure 17. The spatial distribution of the biomarkers across the patients. The level of biomarker expression was shown for all the tumor sectors. Source data are provided as a Source Data file.

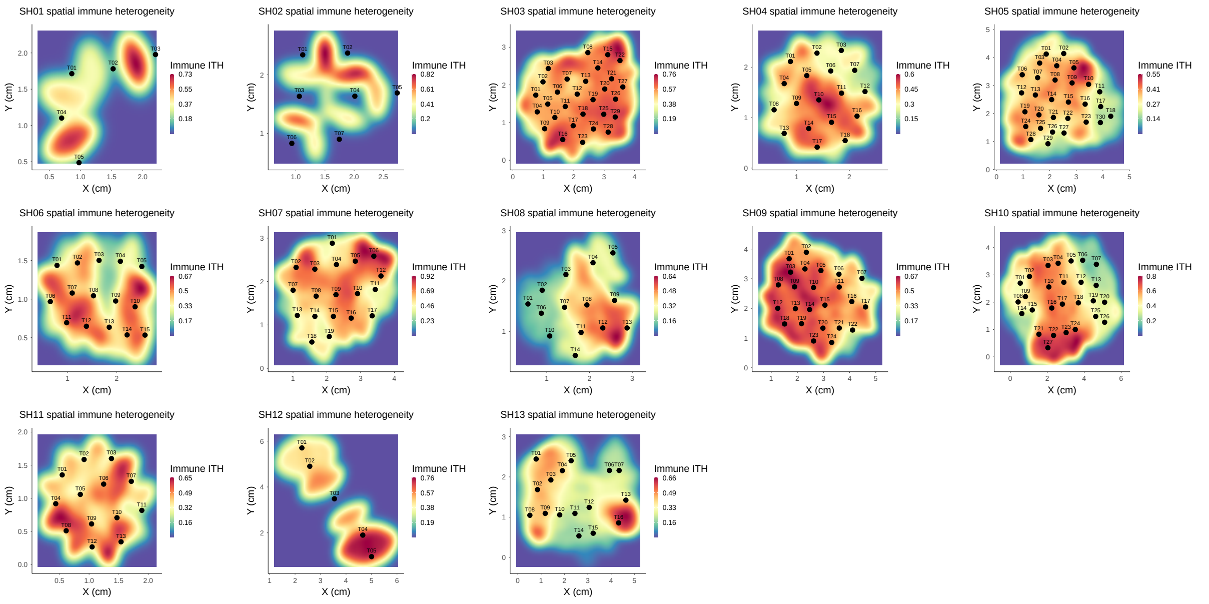

Supplementary Figure 18. Immune heterogeneity across the patients. The immune ITH across the 13 patients. The same style of plot as Figure 2e.

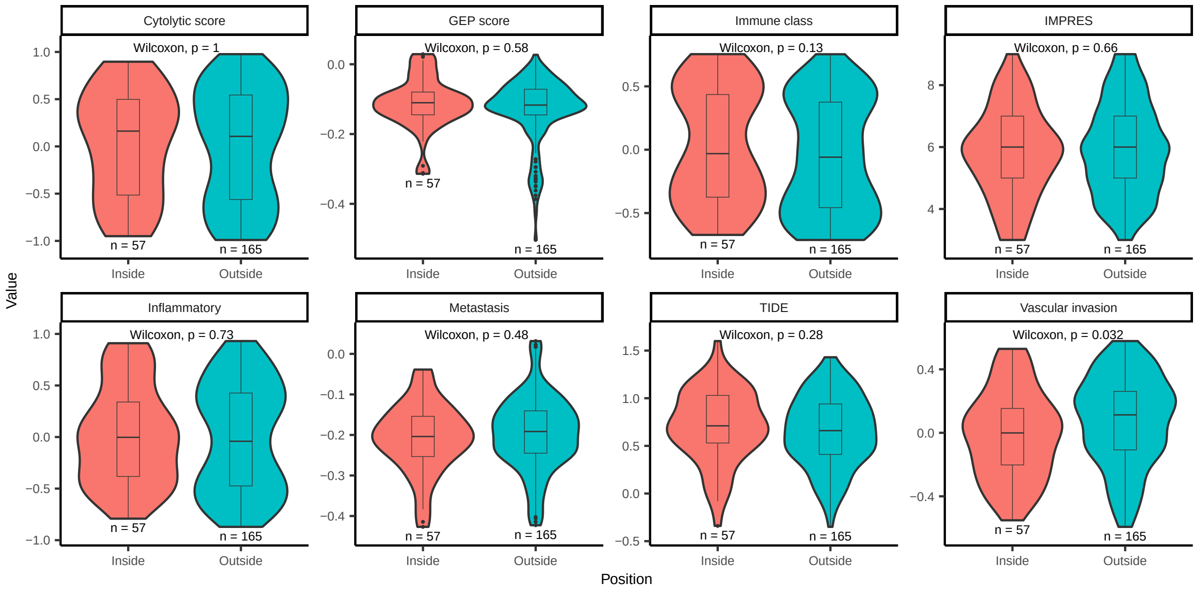

Supplementary Figure 19. Differences in the expression of the biomarkers between central and peripheral regions of the tumor. The lower and upper hinges represent the 25th and 75th percentiles, respectively. Whiskers extend up to 1.5 * IQR from the hinges. Source data are provided as a Source Data file.

Supplementary Note 1: isolation by distance relationship is robust to the distance metrics

In the main-text, we observed a positive correlation between the genetic distance and physical distance known as the isolation by distance (IBD) relationship. As genetic distance is only one of many possible metrics measuring the genetic distance. In this section, we will explore the robustness of the IBD relationship using alternative measurements.

a) Clonal distance

Using PyClone, we have inferred the clonal composition of different sectors. Using Euclidian distance which measures the distance in the clonal composition between multiple sectors, we calculated the relationship between the physical distance and clonal distance. We found a strong IBD relationship between the physical distance and clonal distance (Supplementary Figure 4).

b) Phylogenetic distance

Using the mutation pattern, we can infer the phylogenetic relationship among the samples. For example, we found that using distance methods such as the neighbor-joining method or maximum parsimony, we can infer the phylogenetic relationship among the samples. We found that the phylogenetic relationships inferred from different methods are highly similar (See Supplementary Note 3). Using the phylogenetic distances (e.g. based on maximum parsimony), we can compute the relationship between phylogenetic distance and physical distance. We also found a strong IBD relationship between phylogenetic distance and physical distance (Supplementary Figure 4).

Taken together, under a wide variety of distance metrics, we observed a consistent IBD relationship between genetic distances and physical distances.

Supplementary Note 2: the IBD relationship at the phenotypic level is robust against confounding factors

We discovered that the phenotypic divergence observed at the transcriptomic level also had an isolation-by-distance relationship. Given that the whole transcriptome consists

of both tumor and microenvironmental components, we wondered how robust is the IBD relationship in light of these confounding factors.

1) Tumor purity

Since tumor purity might vary in different regions and can potentially affect the IBD relationship. We explored two different approaches in addressing this question: a) First of all, we took all our samples and their purity estimates, we identified genes that specifically expressed in tumor cells (i.e. genes with expression positively correlated with tumor purity, see Methods). Using these gene sets, we can calculate the phenotypic divergence between sectors and correlate them with the physical distance, we found that the IBD relationship is quite consistent (Supplementary Figure 5). b) Secondly, we performed multivariate regression between transcriptomic distance (y) and physical distance (x_1) taking into account purity differences (x_2). We found that the linear relationship is consistent in the multivariate model taking into account tumor purity (Supplementary Figure 6).

2) Copy number variation

Since tumor transcriptomic profiles can be strongly affected by local copy number profiles. If copy number profiles have regional variations, it might lead to the IBD relationship. In order to look into this factor, we have taken genes in copy neutral regions (i.e. not affected by copy number alternations) and computed the transcriptomic distance based on these copy neutral genes, we found that the IBD relationship is still consistent (Supplementary Figure 7).

Supplementary Note 3: the phylogenetic relationship is consistent across different methods

Using patterns of mutations, we can infer the phylogenetic relationship among tumor sectors. For example, using the presence and absence of mutations across sectors, we can calculate the hamming distance between tumor samples. Using this distance metric, we can infer the phylogenetic relationship among the tumor sectors (i.e. neighbor joining trees). Similarly, we can also infer the phylogenetic relationship based on maximum parsimony (i.e. parsimony trees, Methods). When we compare the

phylogenetic relationship between different trees, we found that the phylogenetic relationship is highly similar between different methods (Supplementary Figure 9).

Supplementary Note 4: statistical properties in detecting natural selection in tumor evolution

Detecting natural selection is a central topic in Evolutionary Biology and is still in its infancy in tumor evolution. Most of the historical developments in the field have been centered around detecting repeated or convergent changes in putative driver genes. Most of the known driver genes are enriched for driver genes in the early history of tumorigenesis. How to detect ongoing natural selection is still an important and challenging topic facing the community. Given its infancy, we would like to discuss potential gaps in this study and the field.

1) The prevalence of natural selection in HCC.

In the 13 HCC patients, we found that there is varying evidence of natural selection in HCC. However, we did not observe a higher prevalence of natural selection in patients with SCD. We think there are a number of reasons for this observation.

1) SCD patients, are defined by the changes at the phenotypic level. However, the existing methods for detecting natural selection are mainly based on the genetic changes. We still lack proper methods modeling and detect natural selection at the phenotypic level. When we used the Sackin S statistic to test the imbalance of the transcriptomic (RNA) tree, we did rejected neutrality for all SCD patients. 2) The number of patients in SCD and non-SCD is not large, even though we do detect important genetic changes associated with the genetic divergence (for example, we observed much elevated genome copy number in the selected subclone in SH06, Fig.4f). These changes are quite "individualized" and is not statistically significant. 3) Thirdly, we think patients with SCD distribution are exceptional cases of tumor evolution where there are often two dominant sub-populations with large genotypic and phenotypic (mixed transcriptomic subtypes) differences. From our limited explorations, we found that the signal of natural selection can be very weak, when we sample within the subpopulations. Patient not in the SCD distribution might represent cases where natural selection is still ongoing and

has not yet yield large phenotypic differences. 4) The number of sectors sampled for each patient varies a lot, this can significantly influence the power of detecting natural selection.

2) Statistical properties of the methods for detecting natural selection

Even though, a number of statistical methods (e.g. MOBSTER) have been developed in the field, their statistical properties haven't been examined very carefully. For example, the imbalance tree test can be heavily influenced by the number of sectors collected for the patient. This can significantly contribute to the fact that we did not observe a higher prevalence of natural selection in patients with SCD distribution. In addition, the driver events for natural selection might not be limited to point mutations. Copy number variations can be an important driving event and it remains unknown how the current methods might behave in this setting. Taken together, studying statistical properties of the neutrality tests deserves a separate study in the coming future.
