# Supplementary material for: Tumor phylogeography reveals block-shaped spatial heterogeneity and the mode of evolution in Hepatocellular Carcinoma
Source: Nat Commun. 2024 Apr 12;15:3169. doi: 10.1038/s41467-024-47541-9 (PMC11015015; doi:10.1038/s41467-024-47541-9)
Supplement: Supplementary file 3 — Description of Additional Supplementary Files [file 41467_2024_47541_MOESM3_ESM.pdf]

<u>Description of Additional Supplementary Files</u>

Supplementary Data 1: Patients' clinical information of this study.

Supplementary Data 2: The physical position of tumors and the basic sequencing information of samples.

Supplementary Data 3: The biomarkers used in this study.
